# Theory-guided design of hydrogen-bonded cobaltoporphyrin frameworks for highly selective electrochemical H_2_O_2_ production in acid

**DOI:** 10.1038/s41467-022-30523-0

**Published:** 2022-05-17

**Authors:** Xuan Zhao, Qi Yin, Xinnan Mao, Chen Cheng, Liang Zhang, Lu Wang, Tian-Fu Liu, Youyong Li, Yanguang Li

**Affiliations:** 1 Institute of Functional Nano & Soft Materials (FUNSOM), Jiangsu Key Laboratory for Carbon-Based Functional Materials and Devices, Soochow University, Suzhou, 215123 China; 2 State Key Laboratory of Structural Chemistry, Fujian Institute of Research on the Structure of Matter, Chinese Academy of Sciences, Fuzhou, 350002 Fujian China; 3 University of the Chinese Academy of Sciences, Beijing, 100049 China; 4 Macao Institute of Materials Science and Engineering (MIMSE), MUST-SUDA Joint Research Center for Advanced Functional Materials, Macau University of Science and Technology, Taipa, 999078 Macao China

**Keywords:** Electrocatalysis, Electrocatalysis, Computational methods, Heterogeneous catalysis

## Abstract

The pursuit of selective two-electron oxygen reduction reaction to H_2_O_2_ in acids is demanding and largely hampered by the lack of efficient non-precious-metal-based electrocatalysts. Metal macrocycles hold promise, but have been relatively underexplored. Efforts are called for to promote their inherent catalytic activities and/or increase the surface exposure of active sites. In this contribution, we perform the high-throughput computational screening of thirty-two different metalloporphyrins by comparing their adsorption free energies towards key reaction intermediates. Cobalt porphyrin is revealed to be the optimal candidate with a theoretical overpotential as small as 40 mV. Guided by the computational predictions, we prepare hydrogen-bonded cobaltoporphyrin frameworks in order to promote the solution accessibility of catalytically active sites for H_2_O_2_ production in acids. The product features an onset potential at ~0.68 V, H_2_O_2_ selectivity of >90%, turnover frequency of 10.9 s^−1^ at 0.55 V and stability of ~30 h, the combination of which clearly renders it stand out from existing competitors for this challenging reaction.

## Abstract (web summary)

Guided by high-throughput computational screening, we report the preparation of hydrogen-bonded cobaltoporphyrin frameworks and demonstrate the achievement of high activity and selectivity for electrochemical H_2_O_2_ production in acid.

## Introduction

Compared to the well-established anthraquinone oxidation process, the two-electron oxygen reduction reaction (2e-ORR) makes possible the on-site, on-demand H_2_O_2_ production, and has thereby attracted increasing attention^1–3^. Although it is kinetically favorable in alkaline solution^4–6^, 2e-ORR in acidic solution is much more scientifically important and practically applicable^3,7,8^. The state-of-the-art candidates in acids are Pd-Hg and Pt-Hg alloys^9,10^, followed by Au and its alloys^11,12^. They are unfortunately precious and sometimes toxic. The search for non-precious-metal-based candidates has been actively pursued with some encouraging progresses over the past several years^13–19^.

Among potential candidates, metal macrocycles such as porphyrins and phthalocyanines hold great promise, but have been relatively underexplored^14,20–22^. Their catalytic activities can be rationally tuned by changing the metal centers or modifying the macrocyclic ligands^23–25^. The well-defined molecular structures also represent ideal model systems to explore the structure-performance correlation for a range of catalytic processes^26–28^. In spite of great potential, the 2e-ORR performances of metal macrocycles remain largely unsatisfactory^14,21^. This is due to their low site-specific activities, and more importantly, their conjugated planar structures that readily agglomerate via intermolecular π-π stacking to form particles with few accessible surface sites^26^. Possible solutions include optimizing molecular structures to promote the inherent catalytic activity or constructing porous frameworks to increase the surface exposure of active sites^29,30^.

Hydrogen-bonded organic frameworks (HOFs) are a large family of materials that are self-assembled from functional building blocks to form porous two-dimensional (2D) or three-dimensional (3D) frameworks via hydrogen-bond interactions^31,32^. Having the advantages of high crystallinity, great surface areas, and abundant porosity, they have been extensively explored for gas separation and storage, chemical sensing, proton conduction, and optical applications^32–35^. HOFs generally have improved chemical stability than their cousin materials—metal organic frameworks in aqueous solution; the extended hydrogen-bond networks within HOFs may also render them more hydrophilic than covalent organic frameworks, and potentially more advantageous for electrochemical applications. Moreover, compared to popular single-atom catalysts (SACs), HOFs incorporated with transition metal macrocycles have well-defined active sites and present ideal model systems to explore the structure-performance correlation. Unfortunately, there are very few investigations about the electrochemical applications of HOFs.

In this study, we perform the high-throughput computational screening for metalloporphyrins with various metal centers using density functional theory (DFT) calculations, and identify cobalt porphyrin to have the best 2e-ORR activity and selectivity. As guided by the theoretical predictions, we accordingly prepare several hydrogen-bonded metalloporphyrin frameworks with similar topology but different metal cores. The cobaltoporphyrinic HOF demonstrates an optimal performance with H_2_O_2_ selectivity of >90% over a broad potential window and catalytic turnover frequency (TOF) of 10.9 s^−1^ at 0.55 V in acids exceeding those of precious metal benchmarks.

## Results

### High-throughput DFT screening and calculations

**Fig. 1:**
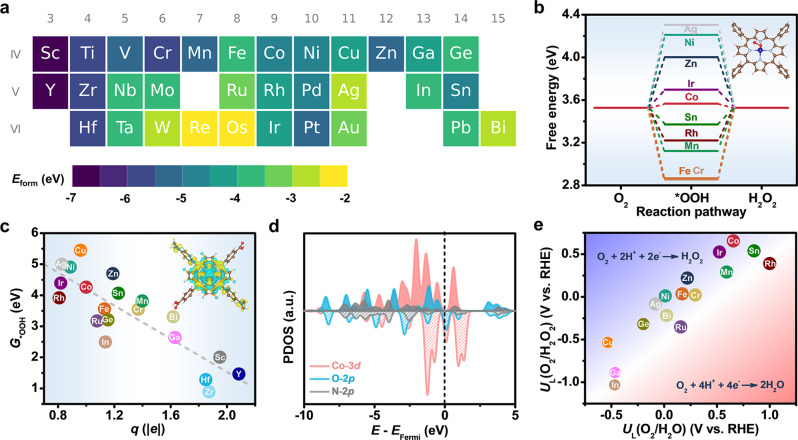
High-throughput DFT screening of different metalloporphyrins. **a** Periodic table with the color of each metal element representing the formation energy of different metalloporphyrins. **b** Free-energy diagram for 2e-ORR at *U* = 0.70 V and (inset) the optimal adsorption configuration of *OOH on cobalt porphyrin. **c** ΔG_*OOH_ as a function of the Bader charge on the metal center of different metalloporphyrins and (inset) the charge density distribution of cobalt porphyrin. **d** PDOS for *OOH adsorbed on cobalt porphyrin. **e** 2D map showing the thermodynamic limiting potential of different metalloporphyrins for 2e-ORR and 4e-ORR.

High-throughput computational screening is an effective method for rapid catalyst exploration and can significantly reduce the time to discovery^36,37^. Here, we constructed metal porphyrins with various metal centers, and compared their structural stability and electrocatalytic performances using high-throughput DFT calculations (see Method for details). The structural model is depicted in Supplementary Fig. S1. Note that the nominal oxidation state of the metal cores is +2 in order to maintain the overall charge neutrality. The formation energy (*E*_form_) is first derived for different metal porphyrins, and summarized in Fig. 1a according to the position of the metals in the periodic table. The negative value of *E*_form_ implies that metal atoms are energetically favorable to form metal porphyrins instead of self-aggregating to clusters. Here, the *E*_form_ values of all the metalloporphyrins are calculated to be negative in the range between −2 and −7 eV, indicating that they are thermodynamically stable for catalytic applications.

We next simulated the ORR process on metal porphyrins via either the two-electron pathway to form H_2_O_2_ or the four-electron pathway to form H_2_O. All the reaction intermediates (e.g., *OOH, *O, and *OH) prefer to adsorb on the metal centers instead of interacting with porphyrin rings or peripheries. Worth noting is that the adsorption free energy of *OOH (ΔG_*OOH_) is an important descriptor for 2e-ORR as first advised by Rossmeisl et al.^9^. Its optimal value is 4.22 eV at *U* = 0 V (or 3.52 eV at *U* = 0.70 V). Figure 1b illustrates the free-energy diagram for 2e-ORR on different metal porphyrins at *U* = 0.70 V. Among all the catalysts investigated, cobalt porphyrin exhibits the best activity with its ΔG_*OOH_ slightly more positive (~0.04 eV) than the ideal value. The inset of Fig. 1b illustrates the corresponding adsorption configuration of *OOH on cobalt porphyrin. The ΔG_*OOH_ value reflects the adsorption strength of *OOH on metal porphyrins, which is closely related to the Bader charge of the metal centers. As shown in Fig. 1c, there is a roughly linear correlation between them: metal centers with more positive charges generally have stronger adsorption strengths towards *OOH. It is worth highlighting that cobalt with the optimal ΔG_*OOH_ value is determined to have a Bader charge of 1.0 *e*. Its charge density distribution is shown in the inset of Fig. 1c. The projected density of states (PDOS) analysis further unveils the electronic properties of cobalt porphyrin (Fig. 1d). The metal center is strongly anchored by nitrogen atoms through the electronic coupling between the N 2p orbital and the Co 3d orbital. Upon the *OOH adsorption, the electronic overlap between the bonded-O 2p orbital of *OOH and the Co 3d orbital is observed, evidencing their direct interaction.

Moreover, we define the thermodynamic limiting potential (*U*_*L*_) as the highest reduction potential where all the reaction steps become downhill in free energy, and use this metric to estimate the relative activities for 2e-ORR and 4e-ORR. A good catalyst would have a positive *U*_*L*_ close to the equilibrium potential of *U*^o^(O_2_/H_2_O_2_) = 0.70 V for 2e-ORR or *U*^o^(O_2_/H_2_O) = 1.23 V for 4e-ORR. Fig. 1e presents the calculated *U*_*L*_(O_2_/H_2_O_2_) and *U*_*L*_(O_2_/H_2_O) values of different metal porphyrins in a 2D map. The reaction selectivity can be readily estimated by comparing the theoretical overpotential (*η* = *U*^o^ – *U*_*L*_) of the two reaction pathways. Catalysts located in the upper left corner have great 2e-ORR selectivity, whereas those located in the lower right corner have great 4e-ORR selectivity. It can be thereby determined that cobalt porphyrin best favors 2e-ORR with *η*(O_2_/H_2_O_2_) = 0.04 V, and that rhodium porphyrin best favors 4e-ORR with *η*(O_2_/H_2_O) = 0.23 V. The combination of great 2e-ORR activity and selectivity of cobalt porphyrin revealed from our high-throughput computations encourages us to experimentally pursue this candidate as would be shown in the following.

### Material synthesis and characterization

**Fig. 2:**
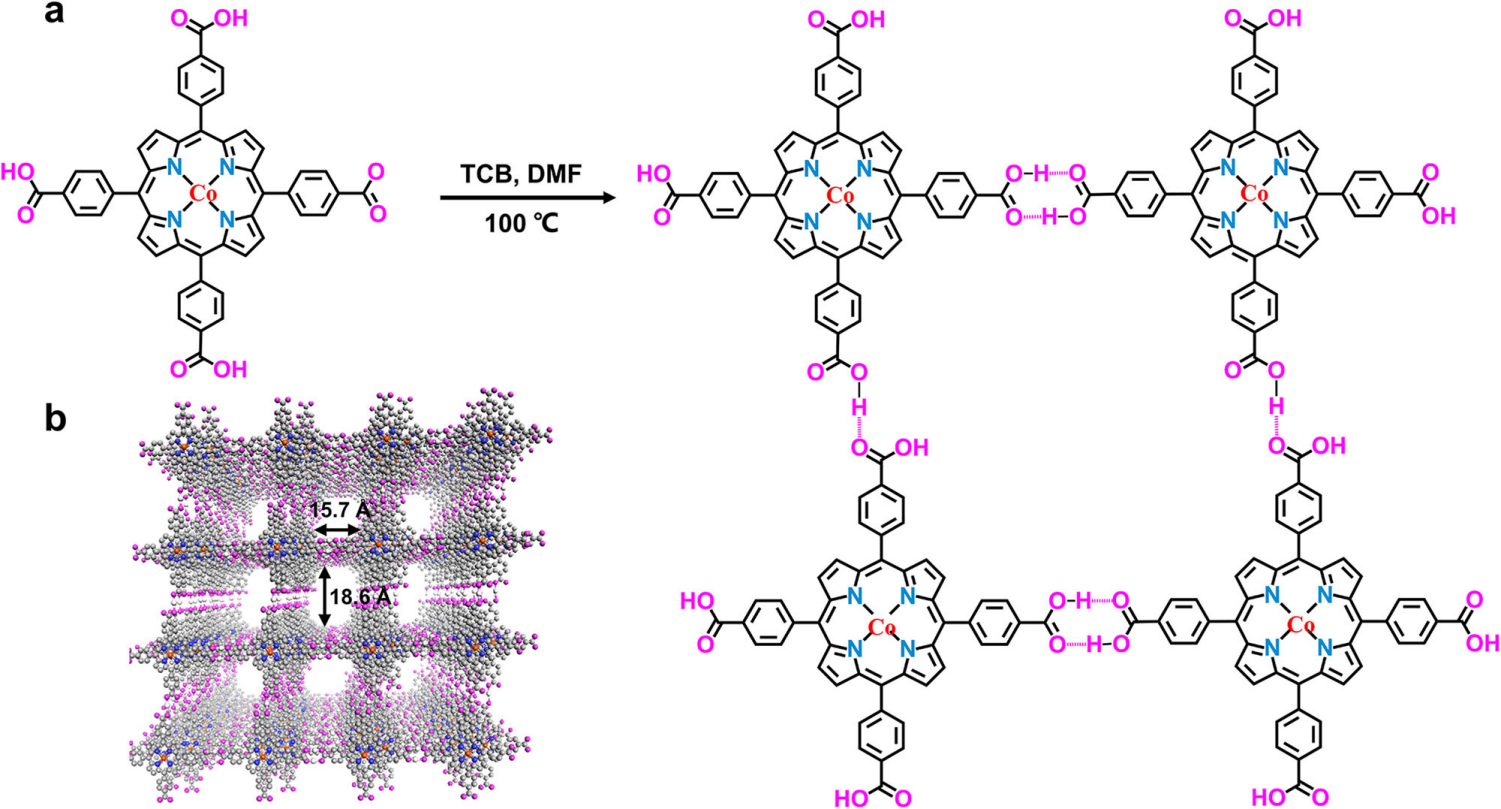
Synthesis and molecular topology of PFC-72-Co. **a** Schematic synthetic procedure toward PFC-72-Co. **b** Schematic topology of PFC-72-Co along the channel direction.

**Fig. 3:**
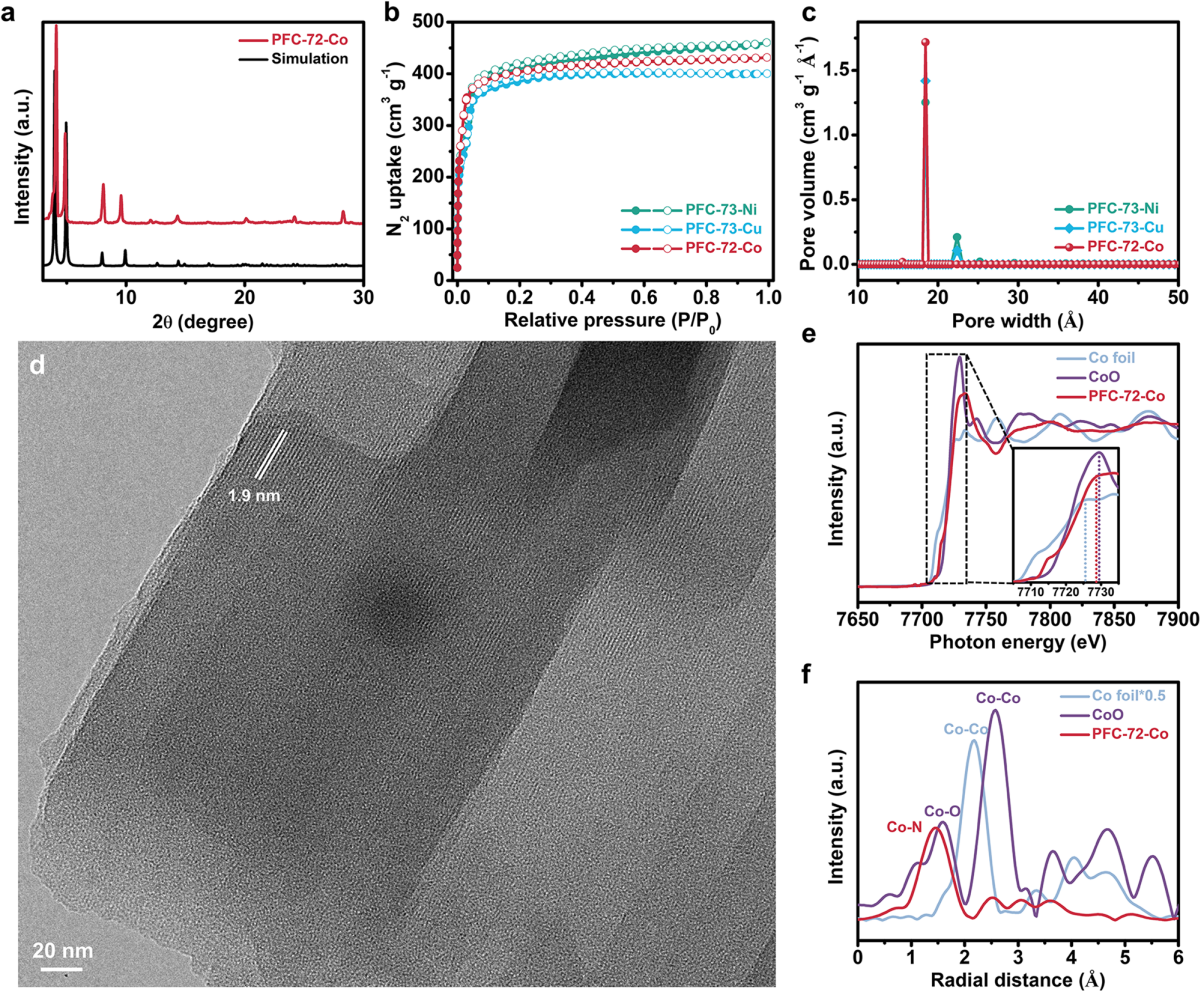
Structural characterizations of PFC-72-Co. **a** PXRD pattern of PFC-72-Co together with its simulation. **b** N_2_ adsorption-desorption isotherms of PFC-72-Co, PFC-73-Ni, and PFC-73-Cu, and **c** their corresponding pore size distribution profiles. **d** TEM image of PFC-72-Co. **e** Co K-edge XANES spectra of PFC-72-Co, the Co foil, and the CoO reference, and **f** corresponding Fourier-transform EXAFS spectra.

Since small-molecule porphyrins tend to agglomerate via intermolecular π-π interactions, we incorporated these functional moieties into porous frameworks. Hydrogen-bonded cobaltoporphyrin frameworks (PFC-72-Co) were prepared through self-assembling 5,10,15,20-(tetra-4-carboxyphenyl) porphyrin (TCPP-Co) in *N,N*-dimethylformamide (DMF) and 1,2,4-trichlorobenzene (TCB) at 100 °C as schematically illustrated in Fig. 2a (see Method for details)^38^. The single-crystal X-ray diffraction (XRD) analysis of PFC-72-Co reveals that it has a 2D square layer topology with opening channels of 18.6 × 15.7 Å^2^ (Fig. 2b and Supplementary Table S1). Individual cobaltoporphyrin unit connects with its four neighbors through the carboxyl groups via O–H···O hydrogen bonds. Adjacent layers are stacked in the AA’ mode with an interlayer distance of 3.62 Å (Supplementary Fig. S2). The porphyrin moiety in PFC-72-Co is experimentally determined to be non-planar and slightly bent as illustrated in Supplementary Fig. S3a. This configuration somewhat deviates from the ideal planar configuration adopted in our DFT calculations but is determined to have little impact on the electrocatalytic activity (Supplementary Fig. S3b). The powdery PXRD pattern of PFC-72-Co exhibits pronounced diffraction peaks in accordance with the standard pattern (simulated based on the crystal structure derived from single-crystal XRD), which evidence its great crystallinity and highly ordered molecular structure (Fig. 3a). The actual Co content in PFC-72-Co measured by inductively coupled plasma – mass spectrometry is close to the theoretical value (Supplementary Table S2). For control experiments, we also prepared isoreticular HOFs based on metal-free porphyrin (PFC-71), nickel porphyrin (PFC-73-Ni), and copper porphyrin (PFC-73-Cu). They have identical topologies but slightly different interlace modes (Supplementary Figs. S2 and S4).

We carried out a multitude of spectroscopic characterizations to confirm the successful preparation of HOFs. The Fourier-transform infrared spectroscopy (FTIR) spectra of metal-free TCPP and PFC-71 are in perfect agreement with previous reports (Supplementary Fig. S5)^39^. Compared to them, the spectra of metalloporphyrins (TCPP-Co/Ni/Cu) and corresponding HOFs (PFC-72-Co, PFC-73-Ni, and PFC-73-Cu) display a new peak around 1000 cm^−1^ (assignable to the stretching vibration of N-M, M = Co, Ni or Cu), whereas the peak around 963 cm^−1^ (assignable to the stretching vibration of N-H) vanishes. The ^1^H nuclear magnetic resonance (NMR) spectra of metal-free TCPP and PFC-71 show the characteristic inner amino proton (N-H) signal at −2.94 ppm, which is however absent in metalloporphyrins and corresponding HOFs (Supplementary Fig. S6), which are consistent with previous reports^40,41^. Raman spectra of TCPP-Co and PFC-72-Co display signature vibration bands of porphyrins as reported in previous works (Supplementary Fig. S7)^42^. The band at 1017 cm^−1^ corresponds to the Co-N stretching vibration. TCPP-Ni/Cu and their corresponding HOFs exhibit similar spectral features as those of TCPP-Co and PFC-72-Co. From the ultraviolet-visible (UV-vis) spectra, all the metalloporphyrins and HOFs exhibit absorption peaks characteristic of porphyrins (Supplementary Fig. S8).

Our products were further characterized by their microstructures. Scanning electron microscopy (SEM) shows that all of them are microsized particles with well-defined shapes and edges (Supplementary Fig. S9). From the broken edges, these particles are observed to consist of stacking laminates. In spite of their slightly different topologies as illustrated in Supplementary Fig. S2, the N_2_ sorption isotherms of our samples reveal comparably high Brunauer–Emmett–Teller surface areas in the range of 1690–1790 m^2^ g^−1^ and prominent channel sizes around ~18.4 Å according to the pore size distribution curves (Fig. 3b, c). Transmission electron microscopy (TEM) image of PFC-72-Co evidences the clear fringes of ~1.9 nm in line with its in-plane lattice spacing (Fig. 3d). Moreover, the coordination environment of the metal center in PFC-72-Co was probed using X-ray absorption (XAS) analysis at the Co K edge. From its X-ray absorption near edge structure (XANES), the white-line peak of PFC-72-Co is observed to locate between those of the Co foil and the CoO reference and much closer to the latter, indicating that the valence state of the Co center in PFC-72-Co is approximately +2 (Fig. 3e). The pre-edge peak at 7715 eV is assignable to the 1s→4p_z_ shakedown transition (inset of Fig. 3e)^43^. The Fourier-transform extended X-ray absorption fine structure (EXAFS) of PFC-72-Co exhibits a major peak at 1.5 Å corresponding to the Co-N coordination (Fig. 3f). The formation of metallic clusters is excluded by the absence of the Co-Co signal at 2.2 Å. All the above spectroscopic and microscopic results unambiguously support the formation of hydrogen-bonded porphyrin frameworks with high crystallinity and metallization of their porphyrin cores.

### Electrochemical performance for 2e-ORR

**Fig. 4:**
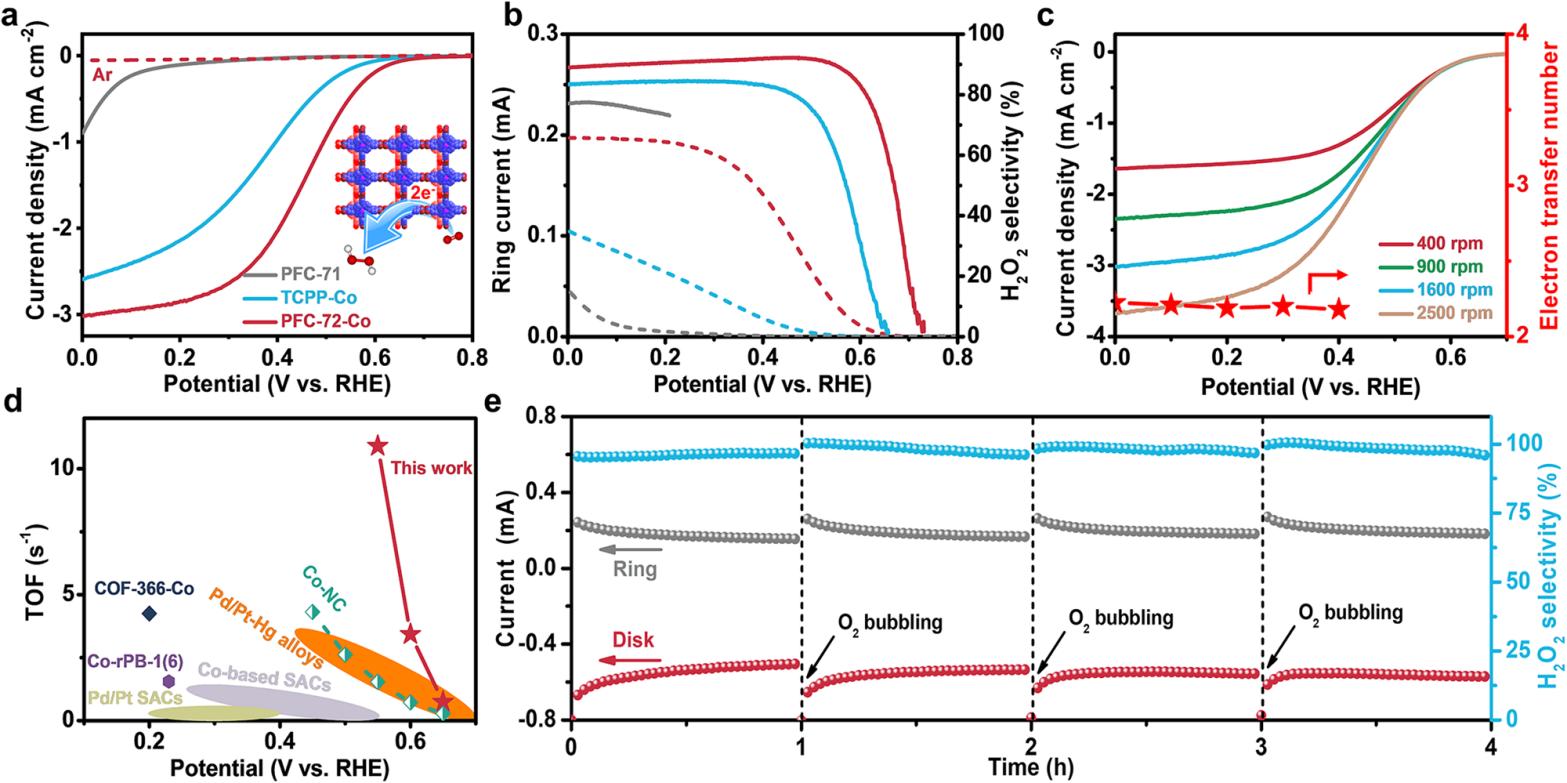
Electrochemical performance of PFC-72-Co for 2e-ORR to H_2_O_2_. **a** Disk polarization curves of PFC-71, TCPP-Co, and PFC-72-Co at 1600 rpm in Ar- or O_2_-saturated 0.1 M HClO_4_. **b** Corresponding ring currents (dash line) and H_2_O_2_ selectivity (solid line) of PFC-71, TCPP-Co, and PFC-72-Co. **c** Disk polarization curves at different electrode rotation speeds and electron transfer numbers derived from the K-L fitting. **d** TOF value of PFC-72-Co in comparison with those of literature results. **e** Changes in the disk current (red), ring current (gray), and H_2_O_2_ selectivity (blue) with time when biased under 0.1 V at 1600 rpm in O_2_-saturated 0.1 M HClO_4_.

We next interrogated the electrochemical performances of PFC-72-Co for 2e-ORR to H_2_O_2_ in 0.1 M HClO_4_. Catalyst powders were loaded onto a rotating ring disk electrode (RRDE) to form smooth thin films with a typical loading of 10 μg cm^−2^ (see Method for details). When the electrolyte is saturated with Ar, no current response is recorded (Fig. 4a). When the electrolyte is saturated with O_2_, metal-free PFC-71 exhibits a negligible cathodic activity until at <0.1 V versus reversible hydrogen electrode (RHE). By stark contrast, the cathodic current density of PFC-72-Co is observed to take off at ~0.68 V (corresponding to ~20 mV overpotential), which is in perfect agreement with the theoretical overpotential of *η* = 40 mV predicted by our DFT calculations. Since the onset, its current density gradually rises and reaches ~3.0 mA cm^−2^ at the working potential of 0 V and the electrode rotation speed of 1600 rpm. The potential-dependent evolution of the disk current density is synchronized with the evolution of the ring current from the electrochemical re-oxidation of H_2_O_2_ on the Pt ring (Fig. 4b). The corresponding H_2_O_2_ selectivity is calculated to be >90% between 0.1 and 0.5 V. Furthermore, PFC-72-Co is assessed at different electrode rotation speeds from 400 to 2500 rpm as shown in Fig. 4c. By fitting J^−1^ ~ ɷ^−1/2^ using the Koutecky–Levich equation (Supplementary Fig. S10), the reaction electron transfer number is estimated to be ~2.2 (Fig. 4c), again corroborating the predominant 2e-ORR selectivity of PFC-72-Co. For comparison, TCPP-Co—in spite of its identical catalytic species with PFC-72-Co—exhibits inferior 2e-ORR activity and selectivity, which highlights the indispensable contribution from the reticular structure of PFC-72-Co during electrocatalysis. PFC-73-Ni and PFC-73-Cu demonstrate even worse activities generally consistent with our theoretical predictions (Supplementary Fig. S11). The electrochemical impedance spectroscopy analysis of PFC-72-Co, TCPP-Co, and PFC-71 unveils that PFC-72-Co has the smallest charge transfer resistance at 0.5 V in line with its greatest electrocatalytic activity (Supplementary Fig. S12). The H_2_O_2_ selectivity of PFC-72-Co (~90%) is jointly contributed by its high production rate and low degradation rate. Negligible cathodic current density is measured on PFC-72-Co during the H_2_O_2_ reduction experiment (Supplementary Fig. S13). In addition, we investigate the effect of the catalyst loading and find that higher loading adversely compromises the H_2_O_2_ selectivity (Supplementary Fig. S14). We also explore the effect of the electrolyte pH and observe that hydrogen bonds in PFC-72 start to dissociate at pH >5 so that it only works in acidic or weakly acidic solution.

In order to elucidate the site-specific activity of PFC-72-Co, we calculated its TOF at different working potentials by normalizing the kinetic current density over the number of electrochemically accessible active sites (Supplementary Fig. S15). It is worth noting that the number of active sites can be estimated by integrating the total charges associated with the Co^II^/Co^III^ oxidation wave during the anodic polarization under N_2_, which is found to be the most reliable method available so far. Fig. 4d depicts the potential-dependent TOF values of PFC-72-Co alongside the typical data of Pt/Pd-Hg alloys^9,10^, Pt/Pd-based SACs^44,45^, Co-based SACs^16,17,46,47^ and Co-based molecules from recent literatures^29,30^. The TOF value of PFC-72-Co is determined to be 0.74 s^−1^ at 0.65 V and 10.9 s^−1^ at 0.55 V, far superior to all of its organic or inorganic competitors as well as the state-of-the-art Pt/Pd-Hg alloys in acids, and underlining its potential for this challenging reaction.

At last, we investigated the potentiostatic stability of PFC-72-Co using RRDE at 1600 rpm in O_2_-satuated 0.1 M HClO_4_. As shown in Fig. 4e, when biased at 0.1 V, the disk and ring currents decline at the beginning and then gradually level off during the first hour. After that, the test is suspended and the electrolyte is bubbled again with O_2_. When the test is resumed, the disk and ring currents go back to the original values, indicating no loss of the catalyst activity. The potentiostatic test can be repeated again and again, and the corresponding H_2_O_2_ selectivity remains >90% during the stability test. Negligible catalyst dissolution or demetallization is observed since the Co concentration of the electrolyte is measured to have no obvious change before and after the test (Supplementary Table S3). Here, we kept the stability test on RRDE relatively short because it is harmful and potentially hazardous for the instrument to continuously rotate for a longer period. The long-term stability of PFC-72-Co was instead pursed by loading the catalyst on a carbon fiber paper electrode in an H-cell. After 30 h operation, the current density remains at ∼5 mA cm^−2^ and the H_2_O_2_ selectivity stays over 85% at 0.1 V (Supplementary Fig. S16). Characterizations of the catalyst after the stability test reveal minimum structural and compositional changes, thereby evidencing its great structural integrity (Supplementary Fig. S17).

## Discussion

In summary, using high-throughput DFT calculations, we screened 32 different kinds of metal porphyrins for 2e-ORR to H_2_O_2_. Among them, cobalt porphyrin was identified to have the optimal activity and selectivity with the theoretical overpotential as low as 0.04 V. Guided by the theoretic predictions, we prepared hydrogen-bonded cobaltoporphyrin frameworks through the self-assembly of TCPP-Co in solution. PFC-72-Co featured high structural crystallinity, large surface areas, and abundant catalytic sites. The product could enable efficient and stable H_2_O_2_ production in 0.1 M HClO_4_ with an onset potential of ~0.68 V and >90% H_2_O_2_ selectivity over a broad potential window. Besides, its TOF value was estimated to be 10.9 s^−1^ at 0.55 V, far superior to its organic or inorganic competitors as well as the state-of-the-art Pt/Pd-Hg alloys in acids. As far as we are aware, our study here represents a demonstration of hydrogen-bonded metalloporphyrin frameworks for electrocatalysis that may expand the potential applications of these interesting materials.

## Methods

### Computational details

The high-throughput computations were performed using DFT as implemented in the Vienna Ab-initio Simulation Package code^48,49^. The projector augmented wave method was used to describe the ion-electron interactions^50,51^. The Perdew–Burke–Ernzerhof functional within the generalized gradient approximation was used to describe the electron exchange–correlation interactions^52,53^. Metal porphyrins with different metal centers were considered as depicted in Supplementary Fig. S1. The structural model contained 91 atoms and had a size of 15 Å × 15 Å. It was placed in a cubic supercell with lattice parameters of 25 Å × 25 Å × 20 Å to minimize the interaction from periodic images. Only Γ-point was adopted for sampling the Brillouin zone. The kinetic energy cutoff for the plane wave was set to be 520 eV. All the geometries were fully optimized until the maximal components of the force on each atom were converged to less than 3 × 10^−2^ eV Å^−1^. Spin-polarization effects were also included throughout the calculations. The calculated electronic energies were converted to free energies by adding zero-point energies and entropic contributions of adsorbates obtained from a harmonic oscillator simulation at 298.15 K. Free-energy corrections for gaseous molecules were summarized in Supplementary Table S4, which were calculated by the ideal gas approximation under the pressure of 101,325 and 3167 Pa for H_2_ and H_2_O, respectively.

The structural stability of metal porphyrins was evaluated from its formation energy (*E*_form_) defined as:

1 $${E}_{{{{{{\rm{form}}}}}}}={E}_{M-P}-{E}_{P}-{E}_{M}$$

where *E*_*M-P*_ and *E*_*P*_ represent the total energies of metal porphyrins and metal-free porphyrins, respectively, and *E*_*M*_ is the energy of metal atom in the bulk phase.

ORR can take place via the four-electron pathway or the two-electron pathway^54–56^. 4e-ORR consists of four reaction steps as shown below:

2 $$\ast +{{{{{{\rm{O}}}}}}}_{2}({{{{{\rm{g}}}}}})+{{{{{{\rm{H}}}}}}}^{+}+{e}^{-}\to \ast {{{{{\rm{OOH}}}}}}$$

3 $$\ast {{{{{\rm{OOH}}}}}}+{{{{{{\rm{H}}}}}}}^{+}+{e}^{-}\to \ast {{{{{\rm{O}}}}}}+{{{{{{\rm{H}}}}}}}_{2}{{{{{\rm{O}}}}}}({{{{{\rm{l}}}}}})$$

4 $$\ast {{{{{\rm{O}}}}}}+{{{{{{\rm{H}}}}}}}^{+}+{{{{{{\rm{e}}}}}}}^{-}\to \ast {{{{{\rm{OH}}}}}}$$

5 $$\ast {{{{{\rm{OH}}}}}}+{{{{{{\rm{H}}}}}}}^{+}+{e}^{-}\to \ast +{{{{{{\rm{H}}}}}}}_{2}{{{{{\rm{O}}}}}}({{{{{\rm{l}}}}}})$$

2e-ORR consists of two reaction steps as shown below:

6 $$\ast +{{{{{{\rm{O}}}}}}}_{2}({{{{{\rm{g}}}}}})+{{{{{{\rm{H}}}}}}}^{+}+{e}^{-}\to \ast {{{{{\rm{OOH}}}}}}$$

7 $$\ast {{{{{\rm{OOH}}}}}}+{{{{{{\rm{H}}}}}}}^{+}+{e}^{-}\to \ast +{{{{{{\rm{H}}}}}}}_{2}{{{{{{\rm{O}}}}}}}_{2}({{{{{\rm{l}}}}}})$$

where * denotes the adsorption site, and *OOH, *O, and *OH denote different adsorbed intermediates. For each step, the Gibbs free energy Δ*G* under the given potential *U* relative to RHE was calculated using the following expression:

8 $$\Delta G=\Delta E+\Delta {{{{{\rm{ZPE}}}}}}-T\Delta S+eU$$

where Δ*E* is the total energy difference between the reactant and product during the reaction, and ΔZPE and *T*Δ*S* are the changes in the zero-point energy and the entropic contribution, respectively.

### Material preparation

#### Preparation of 5,10,15,20-tetrakis(4-methoxycarbonylphenyl)porphyrin (TPPCOOMe)

Six gram of pyrrole and 12 g of methyl p-formylbenzoate were added into 100 mL of propionic acid contained in a 500-mL three-neck flask. The reaction solution was refluxed at 140 °C for 12 h in darkness. After cooling down to room temperature, the solid product was collected via suction-filtration in the form of purple crystals.

#### Preparation of [5,10,15,20-tetrakis(4-methoxycarbonylphenyl)porphyrinato]-Co(II) (TPPCOOMe-Co), TPPCOOMe-Ni, and TPPCOOMe-Cu

0.854 g of TPPCOOMe and 3.1 g of CoCl_2_·6H_2_O were dissolved in 100 mL of DMF and refluxed at 140 °C for 8 h. After cooling back to room temperature, the reaction solution was added with 150 mL of H_2_O. The resultant precipitate was filtered and washed with H_2_O twice. It was then re-dissolved in CHCl_3_, and washed with water for three times. The organic layer was dried over anhydrous MgSO_4_ and evaporated to yield red powders (TPPCOOMe-Co). TPPCOOMe-Ni and TPPCOOMe-Cu were prepared by replacing CoCl_2_·6H_2_O with 3.1 g of NiCl_2_·6H_2_O or 2.2 g of CuCl_2_·2H_2_O, respectively.

#### Preparation of [5,10,15,20-tetrakis(4-carboxyphenyl)porphyrinato]-Co(II) (TCPP-Co), TCPP-Ni, TCPP-Cu, and TCPP

0.75 g of as-prepared TPPCOOMe-M (M = Co, Ni, or Cu) was dissolved in 25 mL of tetrahydrofuran (THF) and 25 mL of methanol, and added with 25 mL of 1.88 M KOH aqueous solution. This mixture was refluxed at 85 °C for 12 h. After cooling down to room temperature, THF and methanol were removed via evaporation. The resultant suspension was added with additional water until the complete dissolution of the solid. It was then acidified with 1 M HCl until no further precipitate was detected. The solid product was collected by filtration, washed with water, and dried in a vacuum. Metal-free TCPP was prepared by replacing TPPCOOMe-M with TPPCOOMe.

#### Preparation of PFC-72-Co, PFC-73-Ni, PFC-73-Cu and PFC-71

Twenty milligrams of TCPP-Co was dissolved in 2 mL of DMF contained in a 15-mL uncapped glass bottle. It was added with 4 mL of TCB, and heated at 100 °C for 3 days. The sample was washed with CH_2_Cl_2_ for three times and then soaked in CH_2_Cl_2_ for 96 h. The supernatant was replaced by fresh CH_2_Cl_2_ several times during the process to exchange and remove nonvolatile solvents (TCB and DMF). Finally, the solid product was collected by vacuum filtration at room temperature, and dried at 100 °C for 8 h. PFC-73-Ni, PFC-73-Cu, and PFC-71 were prepared by replacing TCPP-Co with TCPP-Ni, TCPP-Cu, or TCPP, respectively.

#### Structural characterizations

Single-crystal XRD was collected at 100 or 150 K on a Bruker D8 Venture diffractometer using the Mo-K_α_ radiation (λ = 0.71073 Å). Powder PXRD was carried out on a Rikagu Miniflex 600 Benchtop X-ray diffractometer. FTIR spectra were collected on a VERTEX70 series FTIR spectrometer in the ATR mode. ^1^H-NMR spectra were recorded on a Bruker AVANCE III 400 MHz NMR spectrometer. Raman spectra were measured on a Horiba Labram HR800 Raman spectrometer under the 633 nm laser excitation. UV-Vis spectra were collected on a Shimadzu UV-2550 spectrophotometer. SEM images were obtained from a Zeiss Ultra 55 scanning electron microscope. TEM imaging was performed using an FEI TALOS-200X scanning/transmission electron microscope under the accelerating voltage of 200 kV. Gas sorption experiments were conducted using a Micrometrics ASAP 2460 analyzer. Co K-edge XAS spectra were collected in the transmission mode at the beamline 1W1B of the Beijing Synchrotron Radiation Facility (BSRF, China).

#### Electrochemical measurements

0.5 mg of PFC-72-Co (or other catalysts) and 2 mg of carbon black (Ketjen Black) were first dispersed in 990 µL of ethanol and 10 µL of 5 wt% Nafion solution, and ultrasonicated for 1 h to form a homogeneous catalyst link. 5 µL of the catalyst ink was then dropcast onto the glassy carbon disk (0.2475 cm^2^) of a RRDE electrode (catalyst loading ~10 μg cm^−2^), and dried at room temperature. The loadings for other control catalysts were also ~10 μg cm^−2^ unless otherwise stated. Electrochemical experiments were carried out in a standard three-electrode system controlled by a CHI 760 bipotentiostat and with the RRDE working electrode, a saturated calomel reference electrode (SCE), and a graphite rod counter electrode. The electrolyte was Ar- or O_2_-saturated 0.1 M HClO_4_. All the potential readings were measured against SCE and then converted against RHE. No iR compensation was performed throughout our study. RRDE voltammetry was collected at 10 mV s^−1^ under 1600 rpm. The H_2_O_2_ selectivity was estimated from the oxidation current of the Pt ring (held constant at 1.2 V vs. RHE for the oxidation of H_2_O_2_) using the following equation:

9 $$\% ({{{{{{\rm{H}}}}}}}_{2}{{{{{{\rm{O}}}}}}}_{2})=200\cdot \frac{{I}_{r}/N}{{I}_{d}+{I}_{r}/N}$$

where *I*_*r*_ is the ring current, *I*_*d*_ is the disk current, and *N* (~0.33) is the current collection efficiency of the Pt ring calibrated by a standard ferricyanide system.

## Supplementary information

Supplementary Information

## Data Availability

All data supporting the findings of this study are available from the Source Data. Crystallographic data for the structures reported in this article have been deposited at the Cambridge Crystallographic Data Centre, under deposition numbers CCDC 2107777 (PFC-71), 2107780 (PFC-72-Co), and 2107778 (PFC-73-Cu). Copies of the data can be obtained free of charge via https://www.ccdc.cam.ac.uk/structures/. Source Data are provided with this paper.

## Source data

Source Data

## Code availability

The codes developed in this study are available from the authors upon reasonable request.
