# Supplementary material for: Theory-guided design of hydrogen-bonded cobaltoporphyrin frameworks for highly selective electrochemical H2O2 production in acid
Source: Nat Commun. 2022 May 17;13:2721. doi: 10.1038/s41467-022-30523-0 (PMC9114359; doi:10.1038/s41467-022-30523-0)

Supplementary Information

Theory-Guided Design of Hydrogen-Bonded Cobaltoporphyrin Frameworks for Highly Selective Electrochemical H_2O_2 Production in Acid

Xuan Zhao[1†], Qi Yin[2†], Xinnan Mao[1†], Chen Cheng[1], Liang Zhang[1], Lu Wang[1*], Tian-Fu Liu[2,3*], Youyong Li[1,4] and Yanguang Li[1,4*]

[1]Institute of Functional Nano & Soft Materials (FUNSOM), Jiangsu Key Laboratory for Carbon-Based Functional Materials and Devices, Soochow University, Suzhou 215123, China

[2]State Key Laboratory of Structural Chemistry, Fujian Institute of Research on the Structure of Matter, Chinese Academy of Sciences, Fujian Fuzhou 350002, China

[3]University of the Chinese Academy of Sciences, Beijing 100049, China

[4]Macao Institute of Materials Science and Engineering (MIMSE), MUST-SUDA Joint Research Center for Advanced Functional Materials, Macau University of Science and Technology, Taipa 999078, Macao, China

These authors contributed equally: Xuan Zhao, Qi Yin, Xinnan Mao. Correspondence and requests for materials should be addressed to Y.G.L. (yanguang@suda.edu.cn), T.F.L. (tfliu@fjirsm.ac.cn) or L.W. (lwang22@suda.edu.cn).

Table S1. Crystal data of HOFs determined from single-crystal XRD.

Identification code	PFC-71	PFC-72-Co	PFC-73-Cu
CCDC number	2107777	2107780	2107778
Empirical formula	$C_{48}H_{30}N_4O_8$	$C_{48}H_{28}CoN_4O_8$	$C_{48}H_{28}N_4O_8Cu$
Formula weight	790.76	847.67	852.28
Temperature/K	150.01	180.04	179.99
Crystal system	monoclinic	monoclinic	monoclinic
Space group	C2/c	P2/c	P21/c
a/Å	30.8554(19)	18.033(2)	21.1603(16)
b/Å	30.1016(19)	22.096(2)	44.384(3)
c/Å	8.4694(5)	9.1973(10)	8.0224(5)
α/°	90	90	90
β/°	99.046(4)	104.289(7)	93.441(4)
γ/°	90	90	90
Volume/$Å^3$	7768.5(8)	3551.3(7)	7520.9(9)
Z	4	2	4
ρcalcg/cm^3	0.676	0.793	0.753
μ/mm^{-1}	0.047	0.277	0.324
F(000)	1640.0	870.0	1748.0
Crystal size/mm^3	0.2 × 0.1 × 0.1	0.2 × 0.1 × 0.1	0.2 × 0.1 × 0.1
Radiation		MoKα (λ = 0.71073)	
2θ range for data collection/°	4.232 to 50.054	4.362 to 52.734	4.738 to 52.742
Index ranges	-36 ≤ h ≤ 35, -35 ≤ k ≤ 31, -10 ≤ l ≤ 10	-22 ≤ h ≤ 22, -27 ≤ k ≤ 27, -11 ≤ l ≤ 11	-26 ≤ h ≤ 23, -54 ≤ k ≤ 55, -10 ≤ l ≤ 9
Reflections collected	25456	25484	65707
Independent reflections	6841 [R_{int}= 0.0780, R_{sigma}= 0.0731]	7248 [R_{int}= 0.0962, R_{sigma}= 0.0940]	15276 [R_{int}= 0.1115, R_{sigma}= 0.1030]
Data/restraints/parameters	6841/2/272	7248/591/260	15276/0/550
Goodness-of-fit on F^2	1.010	1.128	1.057
Final R indexes [I>=2σ (I)]	R_1=0.0661, wR_2= 0.1956	R_1= 0.1066, wR_2= 0.2976	R_1= 0.1195, wR_2= 0.3120
Final R indexes [all data]	R_1= 0.1128, wR_2= 0.2245	R_1= 0.1643, wR_2= 0.3512	R_1= 0.1555, wR_2= 0.3321
Largest diff. peak/hole / e $Å^{-3}$	0.28/-0.31	1.28/-0.68	1.53/-2.46

Please also see their checkCIF files on the page 24-36 of this Supplementary Information.

Table S2. Co content in PFC-72-Co.

	Theoretical value	Measured value by ICP
Co content (wt%)	6.95	6.25 ±0.3

Table S3. Co concentration in the electrolyte before and after the 4 h stability test on RRDE.

	Before stability test	After stability test
Co concentrations (ppb)	7.33 ±0.11	7.64 ±0.09

The slight difference in the Co concentration is within the experimental error and (if it does reflect the catalyst dissolution in electrolyte) corresponds to <1% catalyst loss during the stability test.

Table S4. Free energy corrections for gas-phase species.

Species	E_{DFT} (eV)	ZPE (eV)	$-TS$ (eV)	G (eV)
H_2O	-14.220	0.568	-0.673	-14.325
H_2	-6.771	0.269	-0.403	-6.905

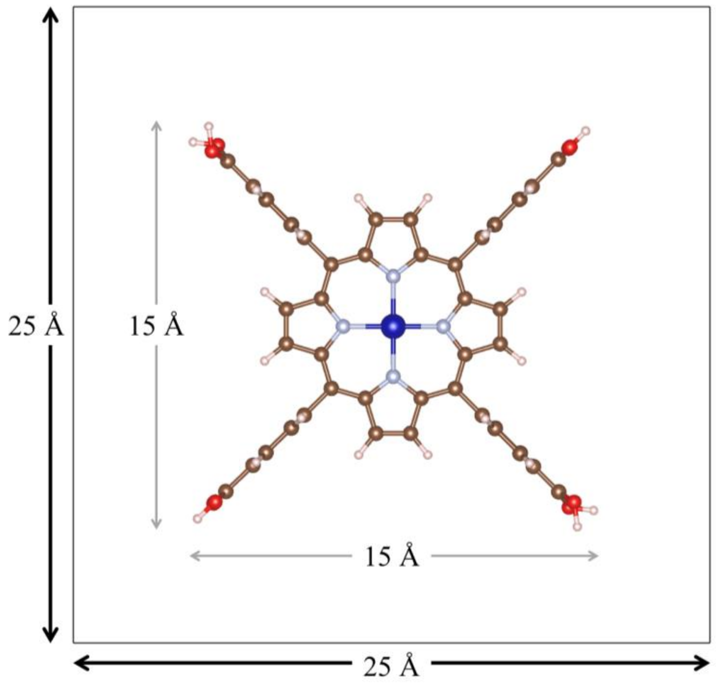

Figure S1. Structural model for our high-throughput DFT calculations. Blue, silver, brown, red and pink spheres represent metal, nitrogen, carbon, oxygen and hydrogen atoms, respectively.

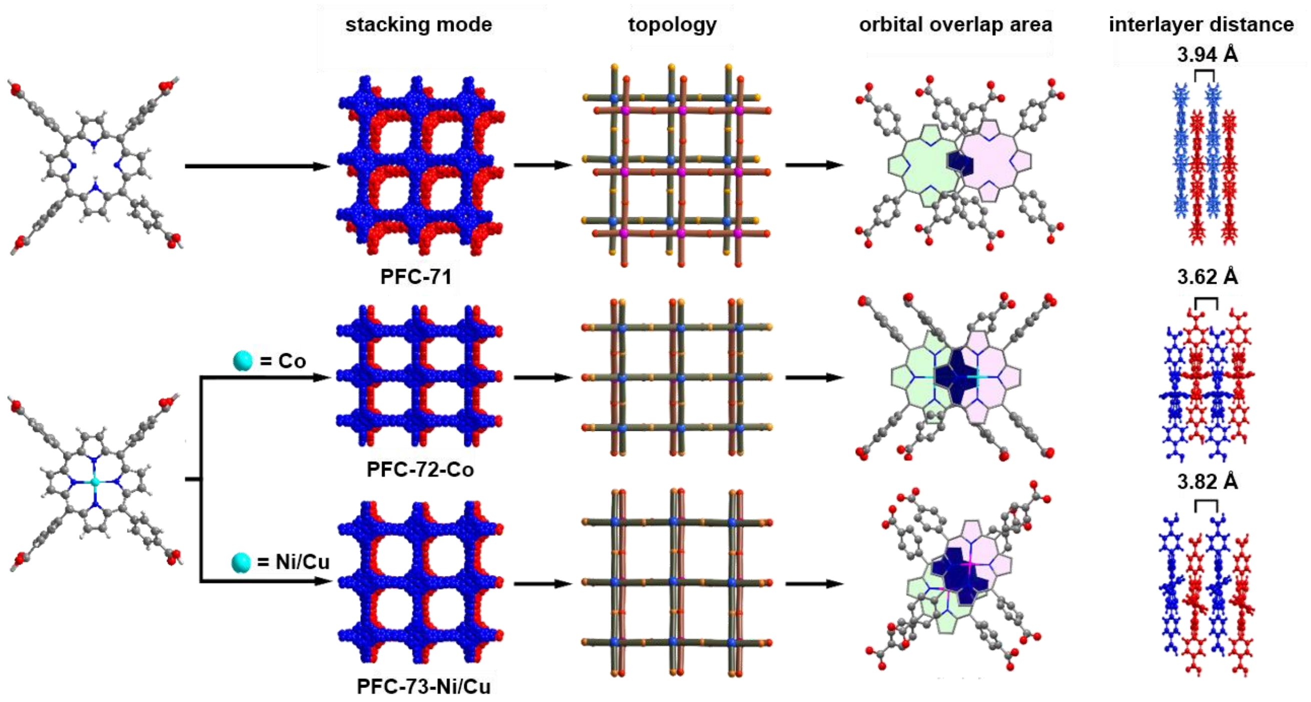

Figure S2. Schematics showing the stacking modes, topologies, orbital overlap areas and interlayer distances for PFC-71, PFC-72-Co, and PFC-73-Ni/Cu.

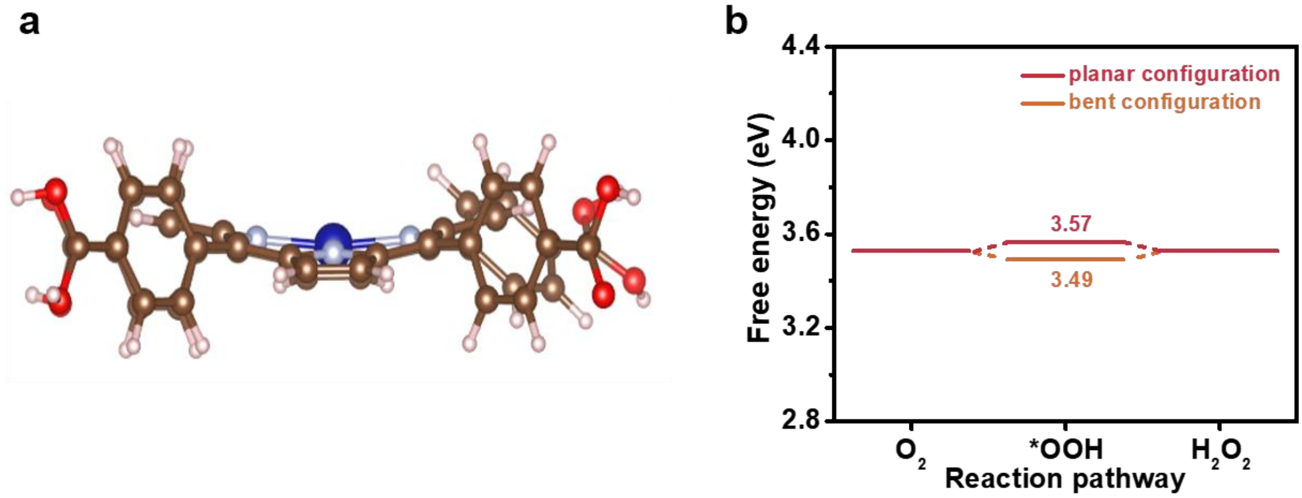

Figure S3. Experimentally determined configuration of cobaltoporphyrin and its adsorption free energy for *OOH. (a) Slightly bent configuration of the cobaltoporphyrin moiety in PFC-72-Co determined from its single-crystal XRD. (b) Adsorption free energy of *OOH on cobaltoporphyrins with the bent configuration or with the planar configuration. Using the experimentally determined molecular configuration, the adsorption free energy of *OOH is calculated to be 3.49 eV, which is only 0.08 eV lower than that calculated with the planar configuration. This slight modification does not change our conclusion about the high activity and selectivity of cobaltoporphyrin towards 2e-ORR.

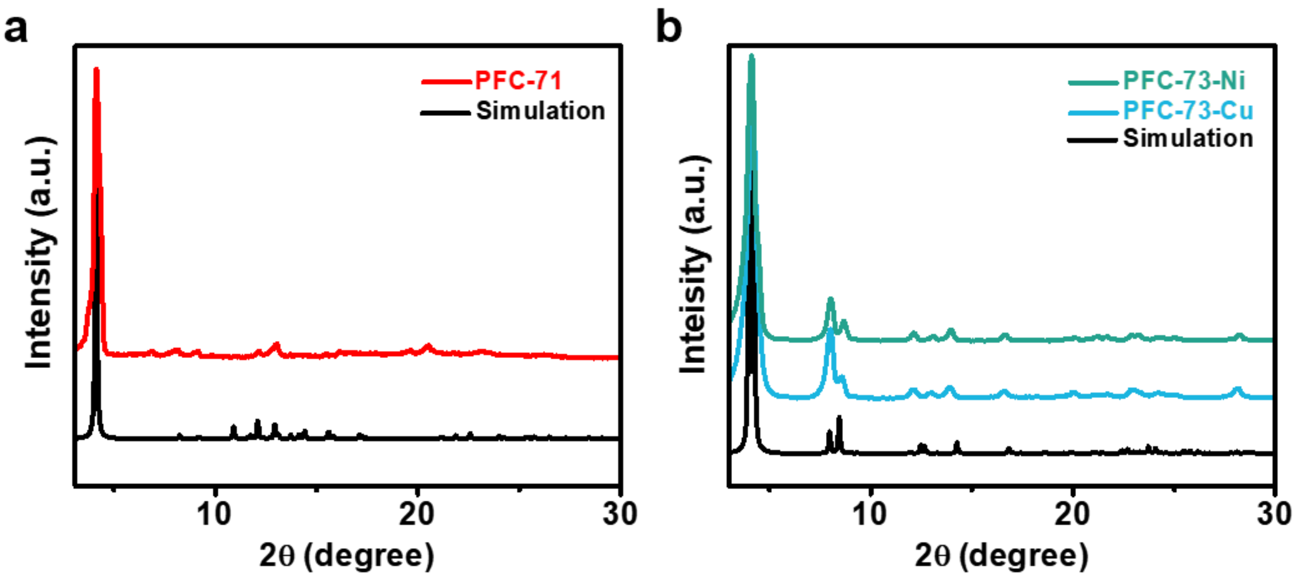

Figure S4. PXRD patterns of HOFs and their simulations. (a) PXRD patterns of metal-free PFC-71 and its simulation. (b) PXRD patterns of PFC-73-Ni, PFC-73-Cu and their simulation.

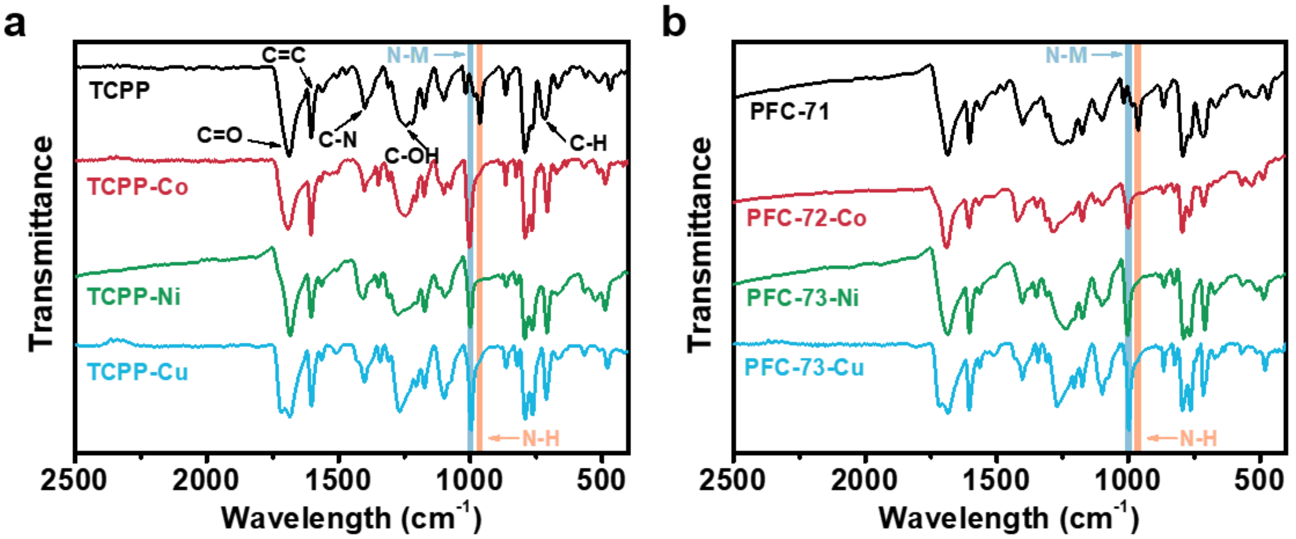

Figure S5. FTIR spectra of porphyrin ligands and their corresponding HOFs. The FTIR spectra of the metal-free TCPP and PFC-71 show the porphyrin fingerprint bands at 1680 cm^{-1}, 1605 cm^{-1}, 1400 cm^{-1}, 1270 cm^{-1} and 710 cm^{-1}, which can be assigned to the stretching vibration of carboxyl C=O, phenyl C=C, pyrrole C-N, carboxyl C-OH, and pyrrole C-H respectively, in perfect agreement with the previous report (*J. Am. Chem. Soc.* **2012**, *134*, 6707). Compared to TCPP and PFC-71, metalloporphyrins (TCPP-Co/Ni/Cu) and corresponding HOFs display a new peak around 1000 cm^{-1} assignable to the stretching vibration of N-M (M = Co, Ni or Cu), whereas the peak around 963 cm^{-1} from the stretching vibration of N-H vanishes. It indicates the successful metallization of the porphyrin cores.

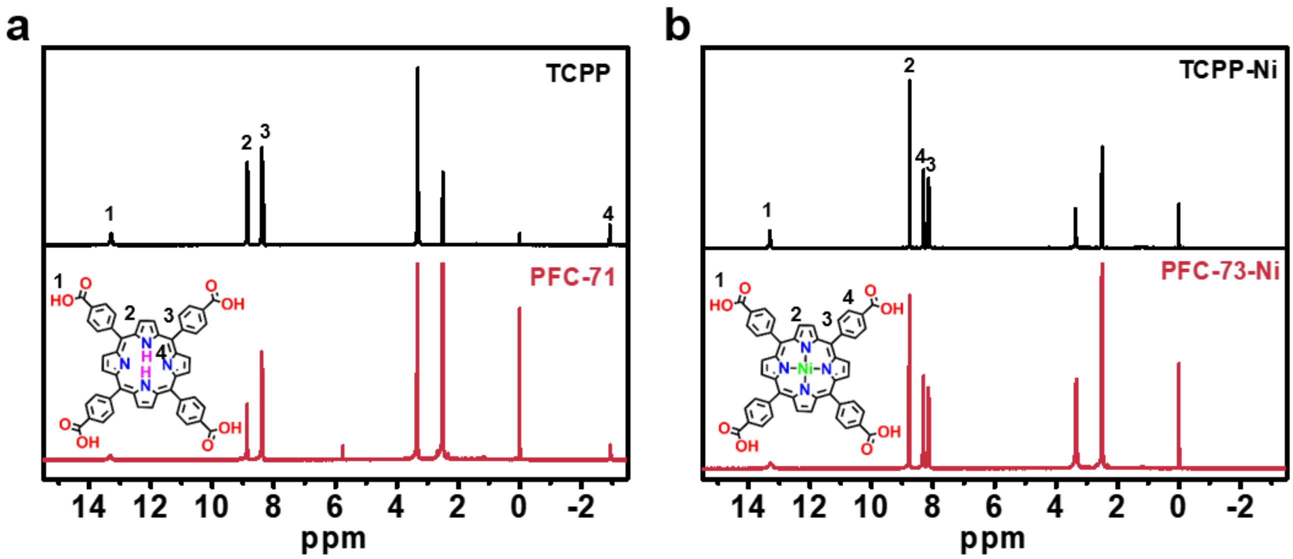

Figure S6. ^{1}H NMR spectra of porphyrin ligands and their corresponding HOFs. (a) ^{1}H NMR spectra of TCPP and PFC-71, ^{1}H NMR (400 MHz, DMSO-d6): δ 13.28 (s, 4H, -COOH, 1), 8.86 (s, 8H, pyrrole C-H, 2), 8.39-8.35 (d, 16H, benzene C-H, 3), -2.94 (s, 2H, pyrrole N-H, 4). (b) ^{1}H NMR spectra of TCPP-Ni and PFC-73-Ni, ^{1}H NMR (400 MHz, DMSO-d6): δ 13.30 (s, 4H, -COOH, 1), 8.76 (s, 8H, pyrrole C-H, 2), 8.32 (d, 8H, benzene C-H, 4), 8.15 (d, 8H, benzene C-H, 3). It should be noted that the NMR spectra of TCPP-Co and PFC-72-Co are not included here because they are strongly perturbed by the magnetism of the Co^{2+} core.

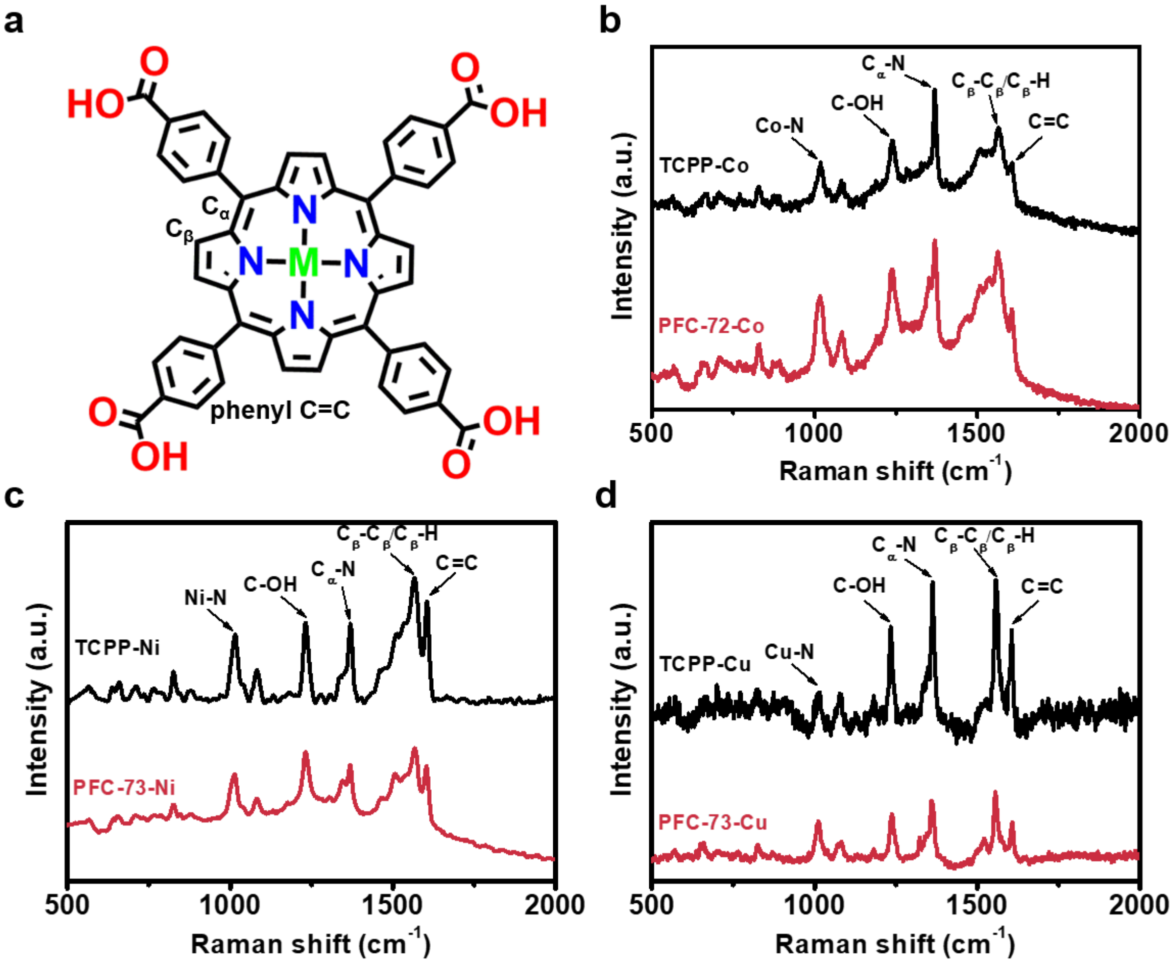

Figure S7. Raman spectra of porphyrin ligands and their corresponding HOFs. Raman spectra of TCPP-Co and PFC-72-Co display signature vibration band of porphyrin at 1270 cm^{-1} (carboxyl C-OH), 1370 cm^{-1} (C_α-N), 1564 cm^{-1} (C_β-C_β and C_β-H) and 1607 cm^{-1} (phenyl C=C). The band at 1017 cm^{-1} is assignable to the Co-N stretching vibration. TCPP-Ni/Cu and their corresponding HOFs exhibit similar spectral features as those of TCPP-Co and PFC-72-Co.

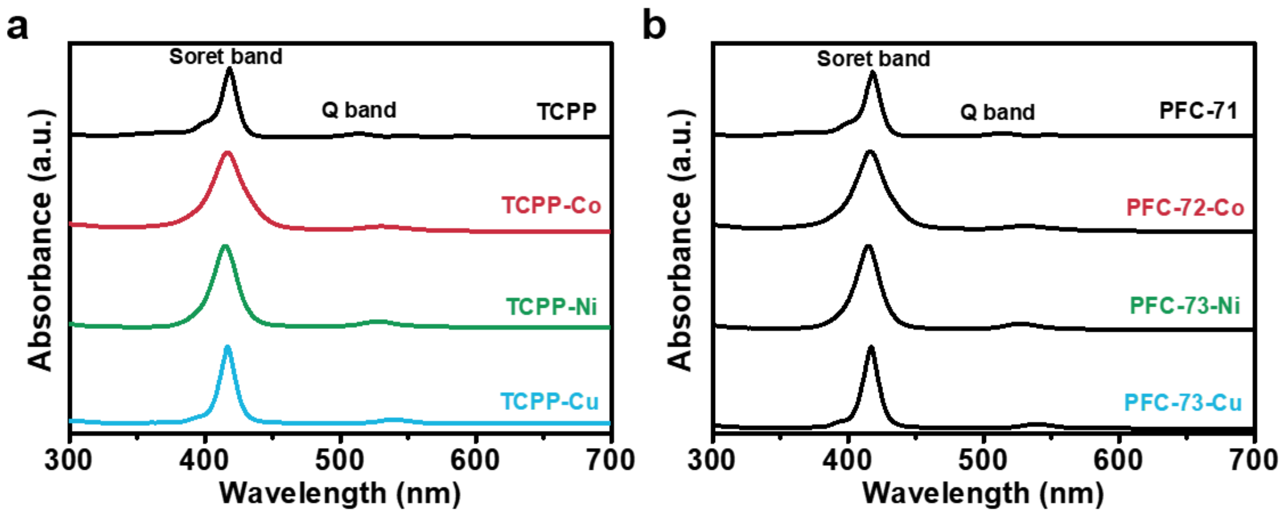

Figure S8. UV-Vis spectra of porphyrin ligands and their corresponding HOFs. UV-Vis measurements were carried out by dissolving sample powders in DMF to achieve a concentration of 0.3 mg mL^{-1}. All the measured spectra exhibit intense Soret bands at ~420 nm and weak Q bands in the range of 500 nm~600 nm. They are characteristic to porphyrins: the Soret bands arise from the transition from the ground state to the second excited state ($S_0 \rightarrow S_2$ π–π*), and Q bands arise from the transition from the ground state to the first excited state ($S_0 \rightarrow S_1$ π–π*) (*J. Chem. Educ.* **1996**, *73*, 1188).

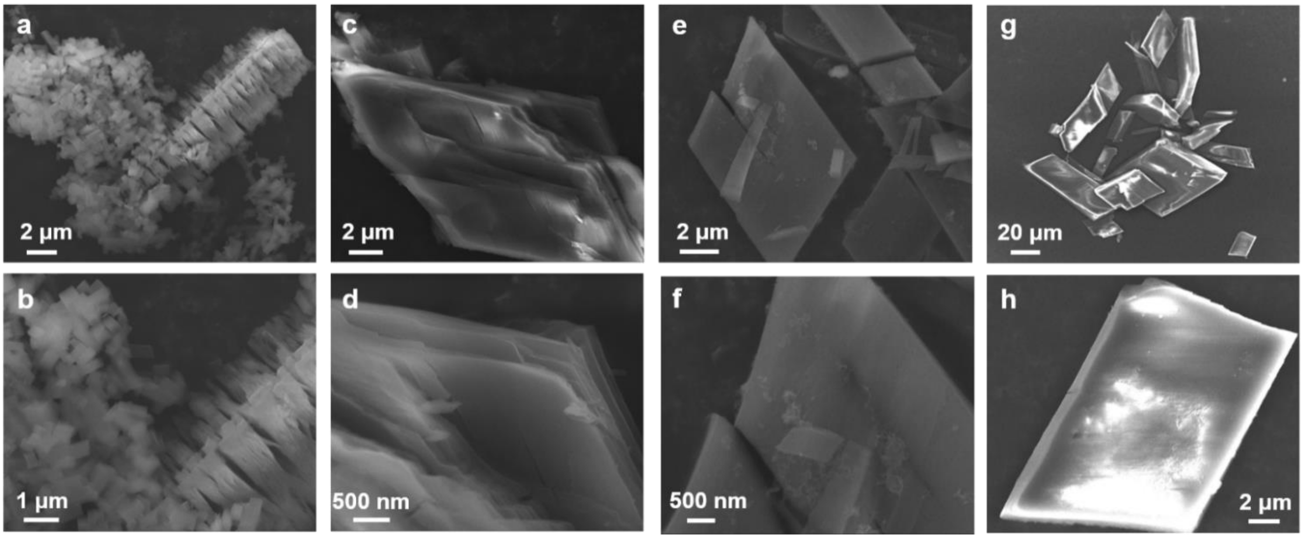

Figure S9. SEM images of different HOFs. (a, b) PFC-71, (c, d) PFC-72-Co, (e, f) PFC-73-Ni and (g, h) PFC-73-Cu.

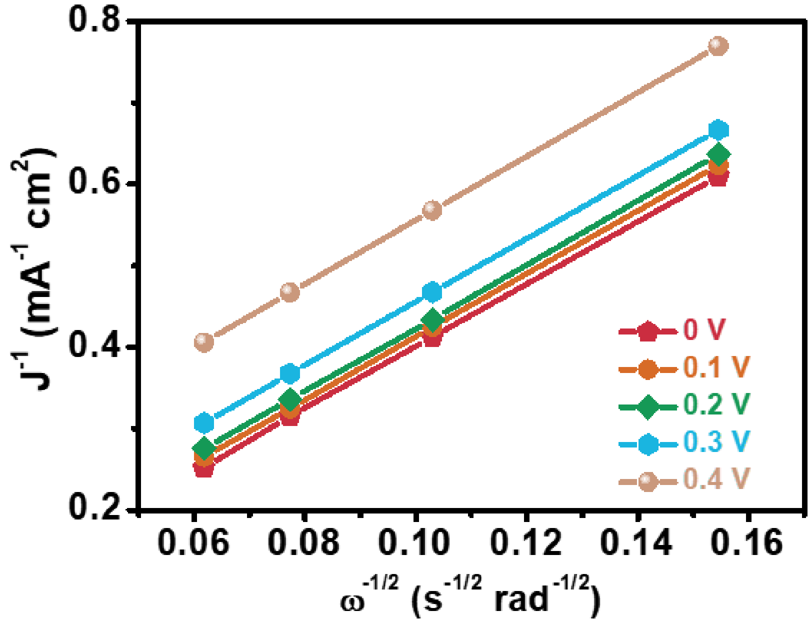

Figure S10. Koutecky-Levich plots of PFC-72-Co at different working potentials in O_2-saturated 0.1 M $HClO_4$.

The Koutecky-Levich equation is as follow:

$$1/J = 1/J_K + 1/J_D = 1/J_K + 1/B\omega_{1/2}$$

$$\omega = 2\pi N$$

$$B = 0.62nFC_oD_o^{2/3}\nu^{-1/6}$$

where J is the current density, J_K and J_D are the kinetic and diffusion-limited current density, ω is the angular velocity, N is the rotation speed, F is the Faraday constant (96485 C mol^{-1}), C_0 is the saturation concentration of O_2 (1.26×10^{-3} mol L^{-1}), D_0 is the diffusion coefficient of O_2 (1.93×10^{-5} cm^2 s^{-1}) and ν is the kinematic viscosity of the electrolyte (0.01 cm^2 s^{-1}) in 0.1 M $HClO_4$. By plotting and fitting $J^{-1} \sim \omega^{-1/2}$ at different working potentials, the slope (corresponding to 1/B) could be derived. As a result, the electron transfer number (n) could be accordingly calculated.

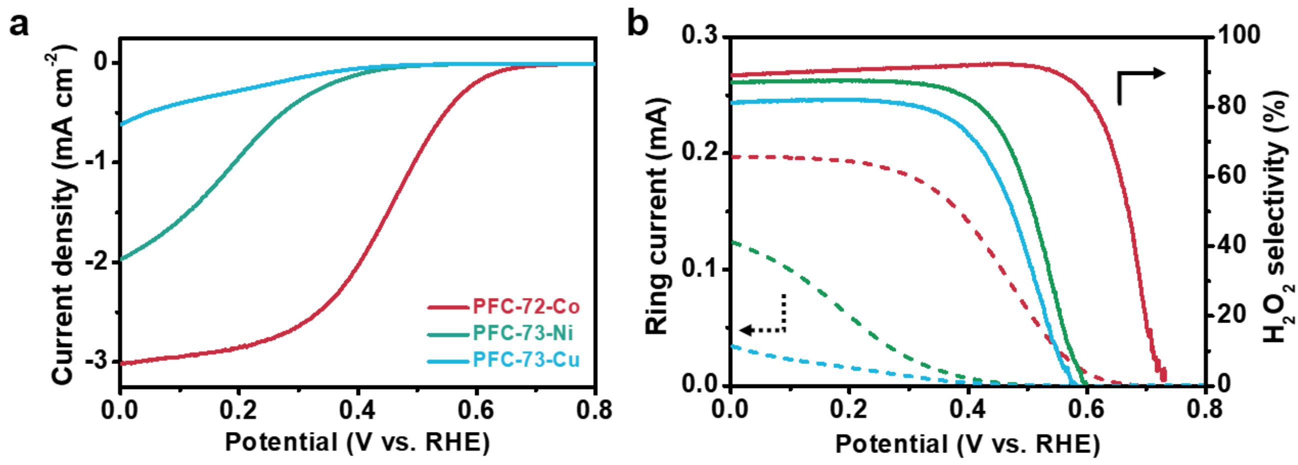

Figure S11. Electrochemical performances of different HOFs. (a) Disk polarization curves of PFC-72-Co, PFC-73-Ni and PFC-73-Cu at 1600 rpm in O_2-saturated 0.1 M $HClO_4$. (b) Their corresponding ring current (dash lines) and H_2O_2 selectivity (solid lines).

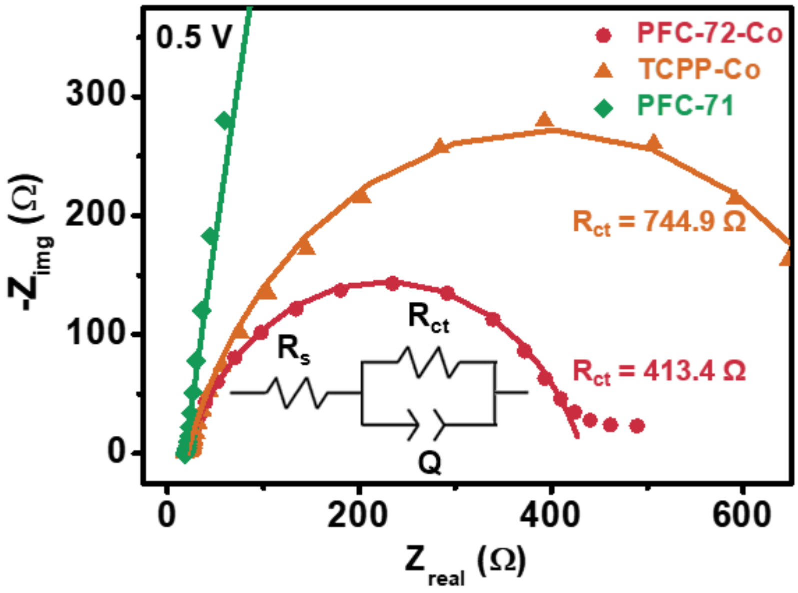

Figure S12. Nyquist plots of PFC-72-Co, TCPP-Co and PFC-71 on RRDE at 1600 rpm and 0.5 V versus RHE in O_2-saturated 0.1 M $HClO_4$ solution. The corresponding equivalent circuit is shown in the inset, where R_s is the solution resistance, R_{ct} is the charge transfer resistance and Q is the constant phase element representing the double-layer capacitance.

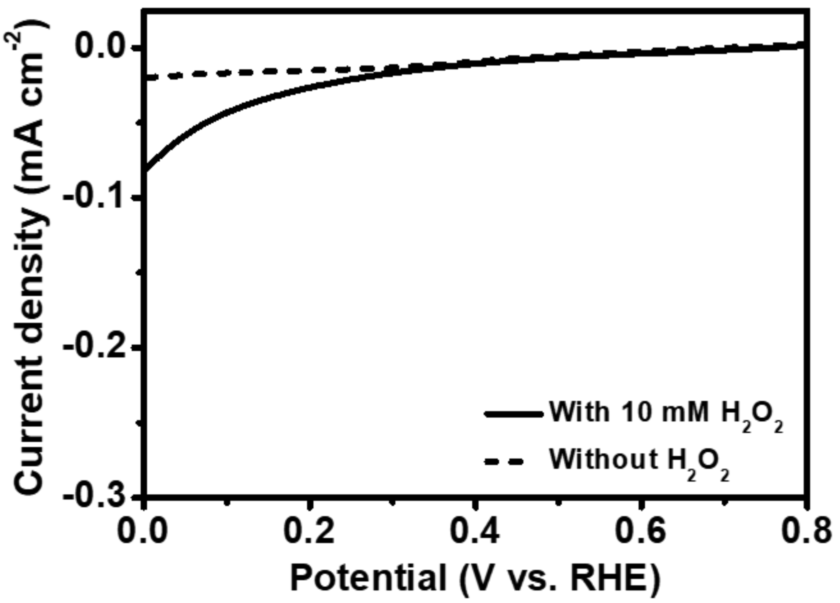

Figure S13. Polarization curves of PFC-72-Co at 1600 rpm in Ar-saturated 0.1 M $HClO_4$ with or without 10 mM H_2O_2. PFC-72-Co only exhibits small cathodic current density of <0.1 mA cm^{-2} in the potential range of 0~0.8 V versus RHE, indicating that it has a poor catalytic activity towards the further reduction of H_2O_2 to H_2O.

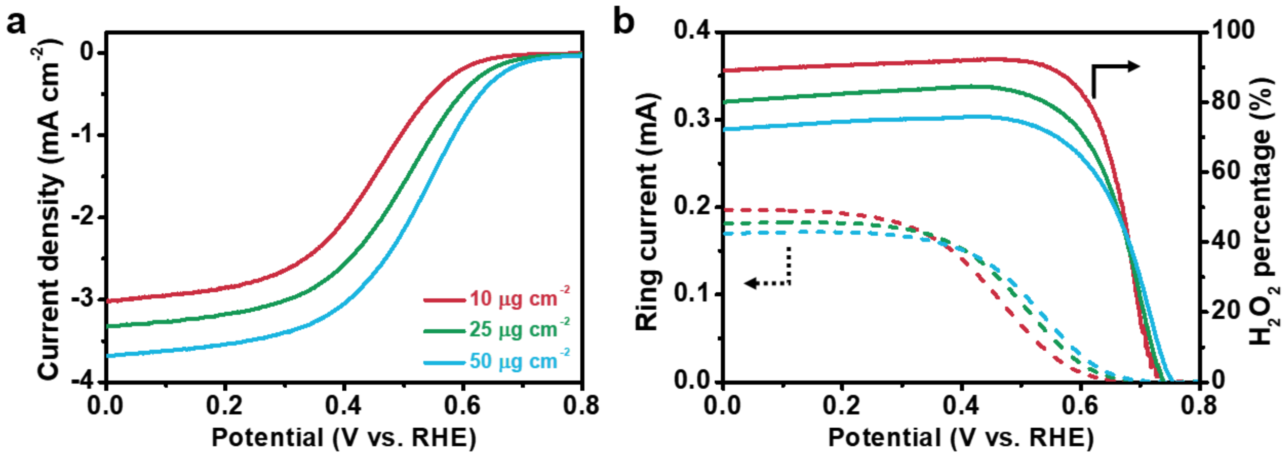

Figure S14. Electrochemical performances of PFC-72-Co with different catalyst loadings. (a) Disk polarization curves of PFC-72-Co with different catalyst loadings. (b) Corresponding ring currents (dash line) and H_2O_2 selectivity (solid line) of PFC-72-Co.

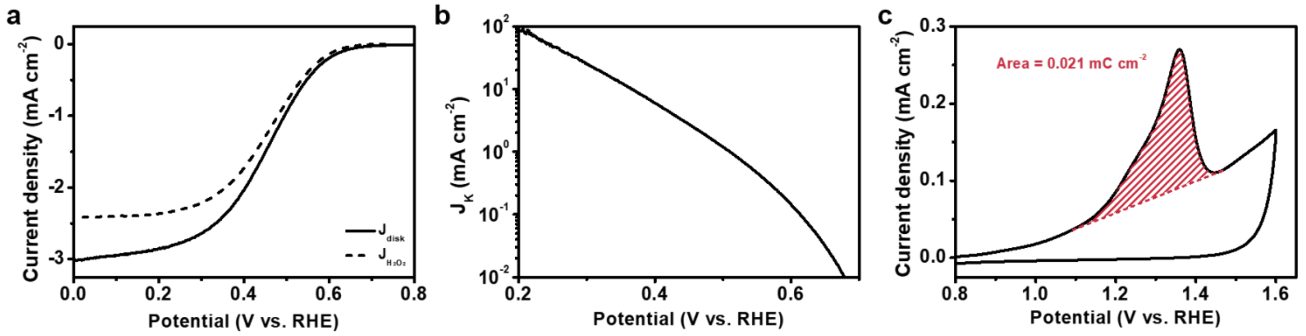

Figure S15. Determination about the potential-dependent TOF value of PFC-72-Co. (a) Disk current density and corresponding H_2O_2 current density of PFC-72-Co at 1600 rpm in O_2-saturated 0.1 M $HClO_4$; (b) corresponding kinetic current density; (c) CV curve of PFC-72-Co in N_2-saturated 0.1 M $HClO_4$.

To derive the potential-dependent TOF value of PFC-72-Co, the H_2O_2 current density (J_{H2O2}) was first calculated by:

$$J_{H_2O_2} = J_{disk} \cdot FE$$

$$FE = \frac{\%(H_2O_2)/100}{2 - \%(H_2O_2)/100}$$

where J_{disk} is the disk current density, FE is the H_2O_2 faradaic efficiency, $\%(H_2O_2)$ is the H_2O_2 selectivity. The kinetic current density (J_K) was then calculated by:

$$\frac{1}{J_K} = \frac{1}{J_{H_2O_2}} - \frac{1}{J_D}$$

where J_D is the diffusion-limited current density (3.025 mA cm^{-2} in 0.1 M $HClO_4$).

In order to estimate the amount of electrochemically accessible Co sites, PFC-72-Co was subjected to CV between 0.8 V~1.6 V (Figure S15c). The anodic wave around 1.3 V corresponded to the one-electron oxidation from Co^{II} to Co^{III}. The total charge associated with this anodic wave was integrated (Q = 0.021 mC cm^{-2}). The amount of surface active Co sites (n_{Co}) was then calculated by:

$$n_{Co} = \frac{Q}{1 \cdot F} = \frac{0.021\ \text{mC cm}^{-2}}{1 \times 96485\ \text{C mol}^{-1}} = 2.177 \times 10^{-7}\ \text{mmol cm}^{-2}$$

Finally, the TOF value was calculated by:

$$\mathrm{TOF} = \frac{J_K}{2 \cdot F \cdot n_{Co}}$$

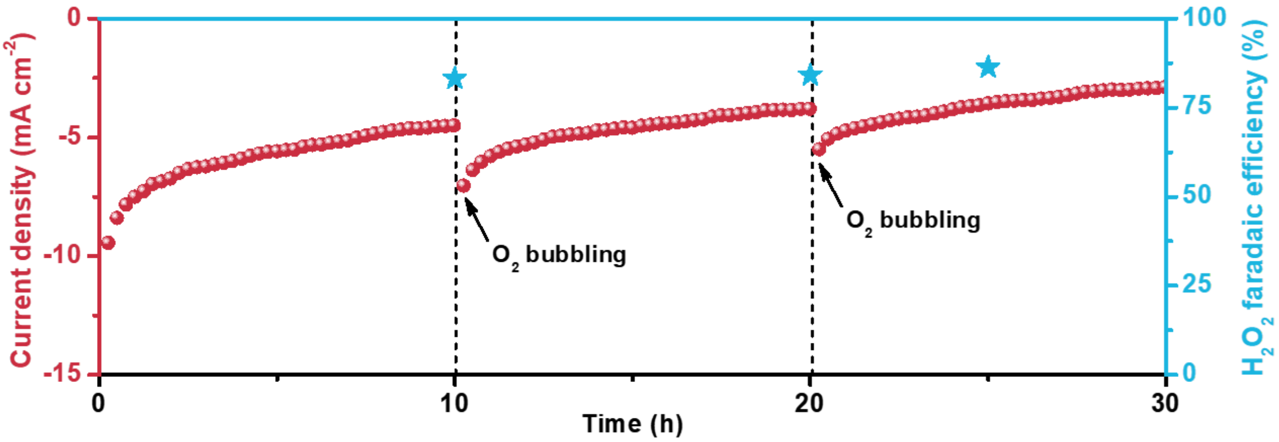

Figure S16. Chronoamperometric (i−t) curve of PFC-72-Co on the carbon fiber paper electrode. To prepare the working electrode, 0.25 mg of PFC-72-Co and 1 mg of carbon black (Ketjen Black) were first dispersed in 250 μL of ethanol and 6.5 μL of 5 wt% Nafion solution, and ultrasonicated for 1 h to form a homogeneous catalyst link. 150 μL of the catalyst ink was then dropcast onto a Teflon-treated carbon fiber paper electrode with an active material loading of 0.15 mg cm^{-2}. The stability was measured under 0.1 V versus RHE in a H-cell filled with O_2-saturated 0.5 M H_2SO_4. The accumulated H_2O_2 concentration in the catholyte was analyzed using the eFOX colorimetric method (*Nano Res.*, **2021**, 10.1007/s12274-021-3882-1) by monitoring the characteristic absorption peak at 556 nm via UV-Vis according to the calibration curve.

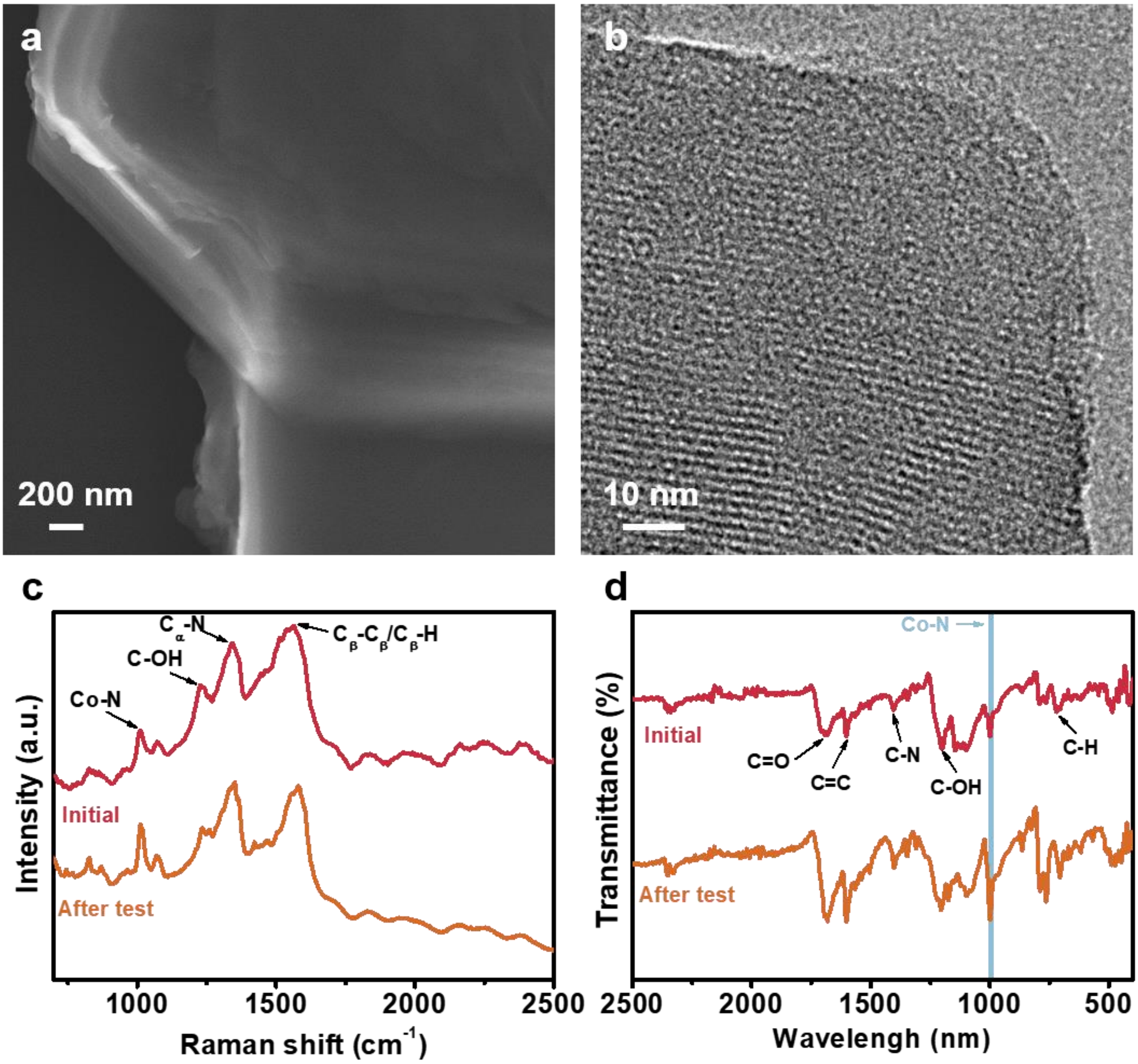

Figure S17. Structural characterizations of PFC-72-Co after the stability test. (a) SEM image and (b) TEM image of PFC-72-Co after the stability test, (c) Raman and (d) FTIR spectra of PFC-72-Co before and after the stability test.

checkCIF/PLATON report

Structure factors have been supplied for datablock(s) pfc-71_sq

THIS REPORT IS FOR GUIDANCE ONLY. IF USED AS PART OF A REVIEW PROCEDURE FOR PUBLICATION, IT SHOULD NOT REPLACE THE EXPERTISE OF AN EXPERIENCED CRYSTALLOGRAPHIC REFEREE.

No syntax errors found. CIF dictionary Interpreting this report

Datablock: pfc-71_sq

Bond precision:	C-C = 0.0035 A		Wavelength=0.71073
Cell:	a=30.8554(19)	b=30.1016(19)	c=8.4694(5)
	alpha=90	beta=99.046(4)	gamma=90
Temperature:	150 K		

	Calculated	Reported
Volume	7768.5(8)	7768.5(8)
Space group	C 2/c	C 1 2/c 1
Hall group	-C 2yc	-C 2yc
Moiety formula	C48 H30 N4 O8 [+ solvent]	C48 H30 N4 O8
Sum formula	C48 H30 N4 O8 [+ solvent]	C48 H30 N4 O8
Mr	790.76	790.76
Dx,g cm-3	0.676	0.676
Z	4	4
Mu (mm-1)	0.047	0.047
F000	1640.0	1640.0
F000'	1640.81	
h,k,lmax	36,35,10	36,35,10
Nref	6869	6841
Tmin,Tmax	0.994,0.995	0.994,0.995
Tmin'	0.991	

Correction method= # Reported T Limits: Tmin=0.994 Tmax=0.995
AbsCorr = MULTI-SCAN

Data completeness= 0.996 Theta(max)= 25.027

R(reflections)= 0.0661(3861) wR2(reflections)= 0.2245(6841)

S = 1.010 Npar= 272

The following ALERTS were generated. Each ALERT has the format
test-name_ALERT_alert-type_alert-level.
Click on the hyperlinks for more details of the test.

Alert level C

```
ABSTY02_ALERT_1_C  An _exptl_absorpt_correction_type has been given without
            a literature citation. This should be contained in the
            _exptl_absorpt_process_details field.
            Absorption correction given as multi-scan
CRYSC01_ALERT_1_C The word below has not been recognised as a standard
            identifier.
            dull
PLAT222_ALERT_3_C NonSolvent Resd 1  H   Uiso(max)/Uiso(min) Range       10.0 Ratio
PLAT250_ALERT_2_C Large U3/U1 Ratio for Average U(i,j) Tensor ....        2.1 Note
PLAT905_ALERT_3_C Negative K value in the Analysis of Variance ...     -8.152 Report
PLAT905_ALERT_3_C Negative K value in the Analysis of Variance ...     -0.220 Report
PLAT911_ALERT_3_C Missing FCF Refl Between Thmin & STh/L=    0.595         24 Report
```

Alert level G

```
PLAT002_ALERT_2_G Number of Distance or Angle Restraints on AtSite          4 Note
PLAT007_ALERT_5_G Number of Unrefined Donor-H Atoms .............           4 Report
PLAT066_ALERT_1_G Predicted and Reported Tmin&Tmax Range Identical          ? Check
PLAT072_ALERT_2_G SHELXL First  Parameter in WGHT  Unusually Large       0.13 Report
PLAT128_ALERT_4_G Alternate Setting for Input Space Group     C2/c       I2/a Note
PLAT169_ALERT_4_G The CIF-Embedded .res File Contains AFIX 1 Recds          2 Report
PLAT172_ALERT_4_G The CIF-Embedded .res File Contains DFIX Records          2 Report
PLAT606_ALERT_4_G Solvent Accessible VOID(S) in Structure ........          ! Info
PLAT720_ALERT_4_G Number of Unusual/Non-Standard Labels ..........         47 Note
PLAT860_ALERT_3_G Number of Least-Squares Restraints .............          2 Note
PLAT869_ALERT_4_G ALERTS Related to the Use of SQUEEZE Suppressed           ! Info
PLAT910_ALERT_3_G Missing # of FCF Reflection(s) Below Theta(Min).          4 Note
PLAT933_ALERT_2_G Number of OMIT Records in Embedded .res File ...         13 Note
PLAT941_ALERT_3_G Average HKL Measurement Multiplicity ..........         3.7 Low
PLAT978_ALERT_2_G Number C-C Bonds with Positive Residual Density.          0 Info
```

0 **ALERT level A** = Most likely a serious problem - resolve or explain
0 **ALERT level B** = A potentially serious problem, consider carefully
7 **ALERT level C** = Check. Ensure it is not caused by an omission or oversight
15 **ALERT level G** = General information/check it is not something unexpected

3 ALERT type 1 CIF construction/syntax error, inconsistent or missing data
5 ALERT type 2 Indicator that the structure model may be wrong or deficient
7 ALERT type 3 Indicator that the structure quality may be low
6 ALERT type 4 Improvement, methodology, query or suggestion
1 ALERT type 5 Informative message, check

It is advisable to attempt to resolve as many as possible of the alerts in all categories. Often the minor alerts point to easily fixed oversights, errors and omissions in your CIF or refinement strategy, so attention to these fine details can be worthwhile. In order to resolve some of the more serious problems it may be necessary to carry out additional measurements or structure refinements. However, the purpose of your study may justify the reported deviations and the more serious of these should normally be commented upon in the discussion or experimental section of a paper or in the "special_details" fields of the CIF. checkCIF was carefully designed to identify outliers and unusual parameters, but every test has its limitations and alerts that are not important in a particular case may appear. Conversely, the absence of alerts does not guarantee there are no aspects of the results needing attention. It is up to the individual to critically assess their own results and, if necessary, seek expert advice.

Publication of your CIF in IUCr journals

A basic structural check has been run on your CIF. These basic checks will be run on all CIFs submitted for publication in IUCr journals (*Acta Crystallographica*, *Journal of Applied Crystallography*, *Journal of Synchrotron Radiation*); however, if you intend to submit to *Acta Crystallographica Section C* or *E* or *IUCrData*, you should make sure that full publication checks are run on the final version of your CIF prior to submission.

Publication of your CIF in other journals

Please refer to the *Notes for Authors* of the relevant journal for any special instructions relating to CIF submission.

PLATON version of 13/07/2021; check.def file version of 13/07/2021

Datablock pfc-71_sq - ellipsoid plot

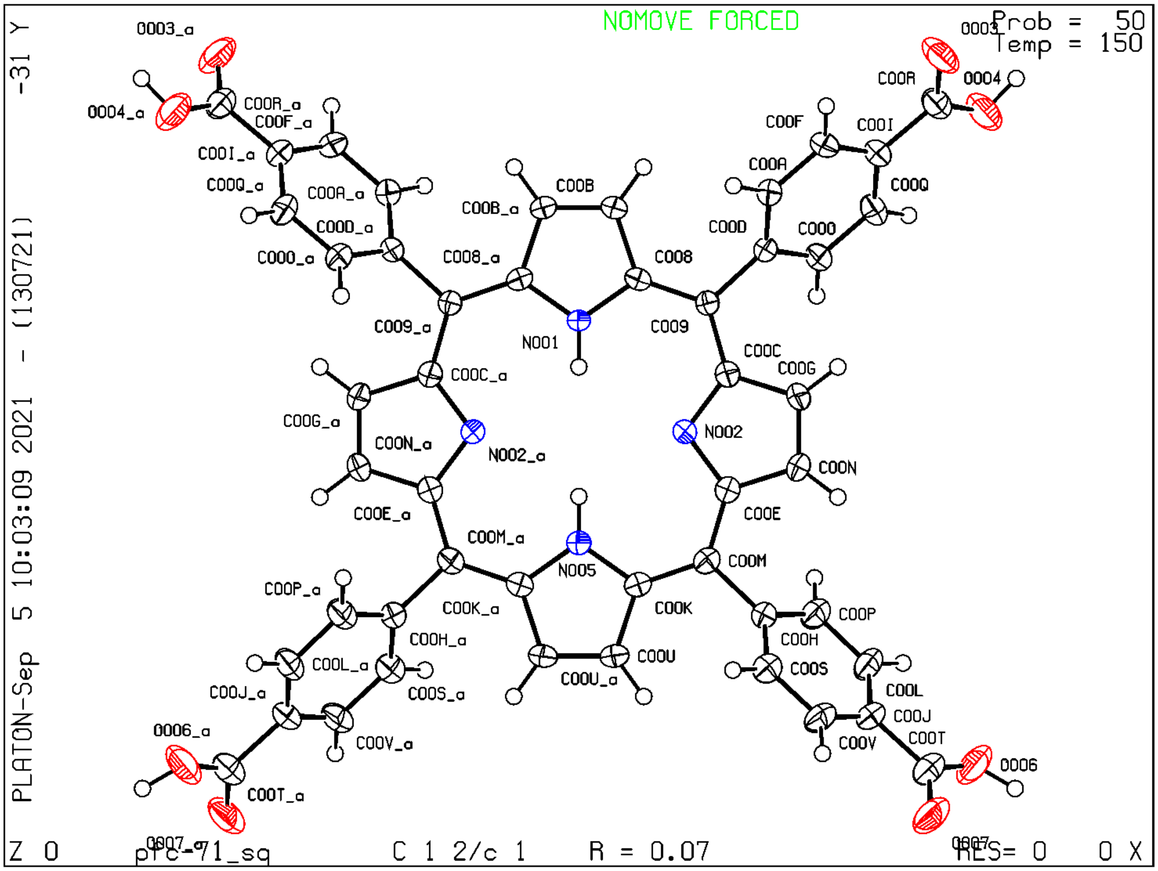

checkCIF/PLATON report

Structure factors have been supplied for datablock(s) pfc-72-co

THIS REPORT IS FOR GUIDANCE ONLY. IF USED AS PART OF A REVIEW PROCEDURE FOR PUBLICATION, IT SHOULD NOT REPLACE THE EXPERTISE OF AN EXPERIENCED CRYSTALLOGRAPHIC REFEREE.

No syntax errors found. CIF dictionary Interpreting this report

Datablock: pfc-72-co

Bond precision:	C-C = 0.0101 A		Wavelength=0.71073
Cell:	a=18.033(2)	b=22.096(2)	c=9.1973(10)
	alpha=90	beta=104.289(7)	gamma=90
Temperature:	180 K		

	Calculated	Reported
Volume	3551.4(6)	3551.3(7)
Space group	P 2/c	P 1 2/c 1
Hall group	-P 2yc	-P 2yc
Moiety formula	C48 H28 Co N4 O8 [+ solvent]	C48 H28 Co N4 O8
Sum formula	C48 H28 Co N4 O8 [+ solvent]	C48 H28 Co N4 O8
Mr	847.67	847.67
Dx,g cm-3	0.793	0.793
Z	2	2
Mu (mm-1)	0.277	0.277
F000	870.0	870.0
F000'	871.08	
h,k,lmax	22,27,11	22,27,11
Nref	7280	7248
Tmin,Tmax	0.967,0.973	0.967,0.973
Tmin'	0.946	

Correction method= # Reported T Limits: Tmin=0.967 Tmax=0.973
AbsCorr = MULTI-SCAN

Data completeness= 0.996 Theta(max)= 26.367

R(reflections)= 0.1066(3781) wR2(reflections)= 0.3512(7248)

S = 1.128 Npar= 260

The following ALERTS were generated. Each ALERT has the format
test-name_ALERT_alert-type_alert-level.
Click on the hyperlinks for more details of the test.

Alert level B

```
PLAT420_ALERT_2_B D-H Bond Without Acceptor  O3       --H3       .     Please Check
```

Author Response: Because of the disorder, we cannot confirm the acceptor. We have dealed with these disorder by squeeze program. Therefore, we believe they have no influence for the refinement and the proposed structure is reliable.

```
PLAT973_ALERT_2_B Check Calcd Positive Resid. Density on       Co1       1.77 eA-3
```

Author Response: We have used two different refienement programs (Shelxl and olex2.refine) to check this alert. Both programs show much smaller residual density on the Co atom. Therefore, we assume that it belongs to the checkcif calculation.

Alert level C

```
ABSTY02_ALERT_1_C  An _exptl_absorpt_correction_type has been given without
            a literature citation. This should be contained in the
            _exptl_absorpt_process_details field.
            Absorption correction given as multi-scan
CRYSC01_ALERT_1_C The word below has not been recognised as a standard
            identifier.
            dull
PLAT082_ALERT_2_C High R1 Value .................................       0.11 Report
PLAT084_ALERT_3_C High wR2 Value (i.e. > 0.25) ..................       0.35 Report
PLAT220_ALERT_2_C NonSolvent   Resd 1  C   Ueq(max)/Ueq(min) Range       5.3 Ratio
PLAT222_ALERT_3_C NonSolvent Resd 1  H   Uiso(max)/Uiso(min) Range       5.8 Ratio
PLAT241_ALERT_2_C High   'MainMol' Ueq as Compared to Neighbors of       C22 Check
PLAT241_ALERT_2_C High   'MainMol' Ueq as Compared to Neighbors of       C23 Check
PLAT241_ALERT_2_C High   'MainMol' Ueq as Compared to Neighbors of       C26 Check
PLAT241_ALERT_2_C High   'MainMol' Ueq as Compared to Neighbors of       C27 Check
PLAT241_ALERT_2_C High   'MainMol' Ueq as Compared to Neighbors of       C28 Check
PLAT242_ALERT_2_C Low    'MainMol' Ueq as Compared to Neighbors of        C3 Check
PLAT242_ALERT_2_C Low    'MainMol' Ueq as Compared to Neighbors of       C15 Check
PLAT242_ALERT_2_C Low    'MainMol' Ueq as Compared to Neighbors of       C20 Check
PLAT242_ALERT_2_C Low    'MainMol' Ueq as Compared to Neighbors of       C21 Check
PLAT250_ALERT_2_C Large U3/U1 Ratio for Average U(i,j) Tensor ....       2.7 Note
PLAT334_ALERT_2_C Small Aver. Benzene C-C Dist C3      -C16_a          1.37 Ang.
PLAT334_ALERT_2_C Small Aver. Benzene C-C Dist C18     -C25_a          1.35 Ang.
PLAT341_ALERT_3_C Low Bond Precision on  C-C Bonds ..............    0.01015 Ang.
```

```
PLAT905_ALERT_3_C Negative K value in the Analysis of Variance ...    -21.784 Report
PLAT905_ALERT_3_C Negative K value in the Analysis of Variance ...     -1.939 Report
PLAT911_ALERT_3_C Missing FCF Refl Between Thmin & STh/L=    0.600        24 Report
PLAT975_ALERT_2_C Check Calcd Resid. Dens.  1.05A   From O1             1.01 eA-3
```

Alert level G

```
PLAT002_ALERT_2_G Number of Distance or Angle Restraints on AtSite          4 Note
PLAT003_ALERT_2_G Number of Uiso or Uij Restrained non-H Atoms ...         28 Report
PLAT007_ALERT_5_G Number of Unrefined Donor-H Atoms .............           3 Report
PLAT066_ALERT_1_G Predicted and Reported Tmin&Tmax Range Identical          ? Check
PLAT072_ALERT_2_G SHELXL First  Parameter in WGHT  Unusually Large       0.20 Report
PLAT169_ALERT_4_G The CIF-Embedded .res File Contains AFIX 1 Recds          3 Report
PLAT171_ALERT_4_G The CIF-Embedded .res File Contains EADP Records          3 Report
PLAT172_ALERT_4_G The CIF-Embedded .res File Contains DFIX Records          3 Report
PLAT178_ALERT_4_G The CIF-Embedded .res File Contains SIMU Records          1 Report
PLAT300_ALERT_4_G Atom Site Occupancy of H1         Constrained at        0.5 Check
PLAT300_ALERT_4_G Atom Site Occupancy of H3         Constrained at        0.5 Check
PLAT606_ALERT_4_G Solvent Accessible VOID(S) in Structure ........          ! Info
PLAT794_ALERT_5_G Tentative Bond Valency for Co1       (II)      .       2.20 Info
PLAT860_ALERT_3_G Number of Least-Squares Restraints ............         591 Note
PLAT910_ALERT_3_G Missing # of FCF Reflection(s) Below Theta(Min).          4 Note
PLAT912_ALERT_4_G Missing # of FCF Reflections Above STh/L=  0.600          4 Note
PLAT913_ALERT_3_G Missing # of Very Strong Reflections in FCF ....          2 Note
PLAT933_ALERT_2_G Number of OMIT Records in Embedded .res File ...          1 Note
PLAT941_ALERT_3_G Average HKL Measurement Multiplicity ..........         3.5 Low
PLAT978_ALERT_2_G Number C-C Bonds with Positive Residual Density.          0 Info
```

---

 0 **ALERT level A** = Most likely a serious problem - resolve or explain
 2 **ALERT level B** = A potentially serious problem, consider carefully
23 **ALERT level C** = Check. Ensure it is not caused by an omission or oversight
20 **ALERT level G** = General information/check it is not something unexpected

```
 3 ALERT type 1 CIF construction/syntax error, inconsistent or missing data
22 ALERT type 2 Indicator that the structure model may be wrong or deficient
10 ALERT type 3 Indicator that the structure quality may be low
 8 ALERT type 4 Improvement, methodology, query or suggestion
 2 ALERT type 5 Informative message, check
```

It is advisable to attempt to resolve as many as possible of the alerts in all categories. Often the minor alerts point to easily fixed oversights, errors and omissions in your CIF or refinement strategy, so attention to these fine details can be worthwhile. In order to resolve some of the more serious problems it may be necessary to carry out additional measurements or structure refinements. However, the purpose of your study may justify the reported deviations and the more serious of these should normally be commented upon in the discussion or experimental section of a paper or in the "special_details" fields of the CIF. checkCIF was carefully designed to identify outliers and unusual parameters, but every test has its limitations and alerts that are not important in a particular case may appear. Conversely, the absence of alerts does not guarantee there are no aspects of the results needing attention. It is up to the individual to critically assess their own results and, if necessary, seek expert advice.

Publication of your CIF in IUCr journals

A basic structural check has been run on your CIF. These basic checks will be run on all CIFs submitted for publication in IUCr journals (*Acta Crystallographica*, *Journal of Applied Crystallography*, *Journal of Synchrotron Radiation*); however, if you intend to submit to *Acta Crystallographica Section C* or *E* or *IUCrData*, you should make sure that full publication checks are run on the final version of your CIF prior to submission.

Publication of your CIF in other journals

Please refer to the *Notes for Authors* of the relevant journal for any special instructions relating to CIF submission.

PLATON version of 13/07/2021; check.def file version of 13/07/2021

Datablock pfc-72-co - ellipsoid plot

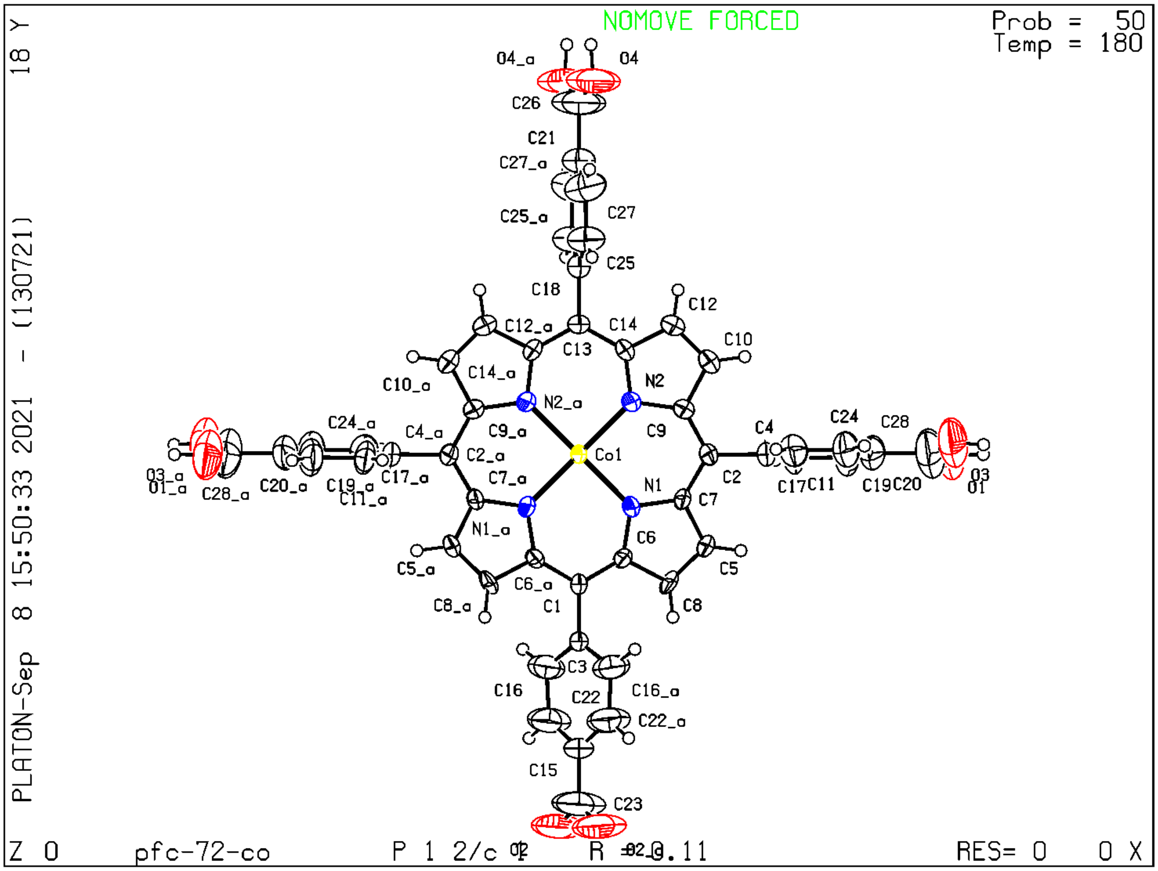

checkCIF/PLATON report

Structure factors have been supplied for datablock(s) pfc-73-cu

THIS REPORT IS FOR GUIDANCE ONLY. IF USED AS PART OF A REVIEW PROCEDURE FOR PUBLICATION, IT SHOULD NOT REPLACE THE EXPERTISE OF AN EXPERIENCED CRYSTALLOGRAPHIC REFEREE.

No syntax errors found. CIF dictionary Interpreting this report

Datablock: pfc-73-cu

Bond precision:	C-C = 0.0089 A		Wavelength=0.71073
Cell:	a=21.1603(16)	b=44.384(3)	c=8.0224(5)
	alpha=90	beta=93.441(4)	gamma=90
Temperature:	180 K		

	Calculated	Reported
Volume	7520.9(9)	7520.9(9)
Space group	P 21/c	P 1 21/c 1
Hall group	-P 2ybc	-P 2ybc
Moiety formula	C48 H28 Cu N4 O8 [+ solvent]	C48 H28 Cu N4 O8
Sum formula	C48 H28 Cu N4 O8 [+ solvent]	C48 H28 Cu N4 O8
Mr	852.29	852.28
Dx,g cm-3	0.753	0.753
Z	4	4
Mu (mm-1)	0.324	0.324
F000	1748.0	1748.0
F000'	1750.04	
h,k,lmax	26,55,10	26,55,10
Nref	15347	15276
Tmin,Tmax	0.962,0.968	0.962,0.968
Tmin'	0.937	

Correction method= # Reported T Limits: Tmin=0.962 Tmax=0.968
AbsCorr = MULTI-SCAN

Data completeness= 0.995 Theta(max)= 26.371

R(reflections)= 0.1195(10046) wR2(reflections)= 0.3321(15276)

S = 1.057 Npar= 550

The following ALERTS were generated. Each ALERT has the format
test-name_ALERT_alert-type_alert-level.
Click on the hyperlinks for more details of the test.

Alert level C

ABSTY02_ALERT_1_C An _exptl_absorpt_correction_type has been given without
a literature citation. This should be contained in the
_exptl_absorpt_process_details field.
Absorption correction given as multi-scan
CRYSC01_ALERT_1_C The word below has not been recognised as a standard
identifier.
dull
DIFMN02_ALERT_2_C The minimum difference density is < -0.1*ZMAX*0.75
_refine_diff_density_min given = -2.456
Test value = -2.175
DIFMN03_ALERT_1_C The minimum difference density is < -0.1*ZMAX*0.75
The relevant atom site should be identified.
PLAT082_ALERT_2_C High R1 Value .... 0.12 Report
PLAT084_ALERT_3_C High wR2 Value (i.e. > 0.25) .... 0.33 Report
PLAT098_ALERT_2_C Large Reported Min. (Negative) Residual Density -2.46 eA-3
PLAT213_ALERT_2_C Atom O6 has ADP max/min Ratio ..... 3.1 oblate
PLAT242_ALERT_2_C Low 'MainMol' Ueq as Compared to Neighbors of C33 Check
PLAT341_ALERT_3_C Low Bond Precision on C-C Bonds .... 0.0089 Ang.
PLAT906_ALERT_3_C Large K Value in the Analysis of Variance ...... 2.031 Check
PLAT910_ALERT_3_C Missing # of FCF Reflection(s) Below Theta(Min). 10 Note
PLAT911_ALERT_3_C Missing FCF Refl Between Thmin & STh/L= 0.600 36 Report
PLAT977_ALERT_2_C Check Negative Difference Density on H6 -0.38 eA-3

Alert level G

PLAT007_ALERT_5_G Number of Unrefined Donor-H Atoms .... 4 Report
PLAT066_ALERT_1_G Predicted and Reported Tmin&Tmax Range Identical ? Check
PLAT072_ALERT_2_G SHELXL First Parameter in WGHT Unusually Large 0.12 Report
PLAT083_ALERT_2_G SHELXL Second Parameter in WGHT Unusually Large 53.63 Why ?
PLAT169_ALERT_4_G The CIF-Embedded .res File Contains AFIX 1 Recds 4 Report
PLAT606_ALERT_4_G Solvent Accessible VOID(S) in Structure .... ! Info
PLAT869_ALERT_4_G ALERTS Related to the Use of SQUEEZE Suppressed ! Info
PLAT912_ALERT_4_G Missing # of FCF Reflections Above STh/L= 0.600 25 Note
PLAT913_ALERT_3_G Missing # of Very Strong Reflections in FCF .... 2 Note
PLAT933_ALERT_2_G Number of OMIT Records in Embedded .res File ... 11 Note
PLAT941_ALERT_3_G Average HKL Measurement Multiplicity .... 4.3 Low
PLAT978_ALERT_2_G Number C-C Bonds with Positive Residual Density. 0 Info

---

0 **ALERT level A** = Most likely a serious problem - resolve or explain
0 **ALERT level B** = A potentially serious problem, consider carefully
14 **ALERT level C** = Check. Ensure it is not caused by an omission or oversight
12 **ALERT level G** = General information/check it is not something unexpected

4 ALERT type 1 CIF construction/syntax error, inconsistent or missing data

```
10 ALERT type 2 Indicator that the structure model may be wrong or deficient
 7 ALERT type 3 Indicator that the structure quality may be low
 4 ALERT type 4 Improvement, methodology, query or suggestion
 1 ALERT type 5 Informative message, check
```

It is advisable to attempt to resolve as many as possible of the alerts in all categories. Often the minor alerts point to easily fixed oversights, errors and omissions in your CIF or refinement strategy, so attention to these fine details can be worthwhile. In order to resolve some of the more serious problems it may be necessary to carry out additional measurements or structure refinements. However, the purpose of your study may justify the reported deviations and the more serious of these should normally be commented upon in the discussion or experimental section of a paper or in the "special_details" fields of the CIF. checkCIF was carefully designed to identify outliers and unusual parameters, but every test has its limitations and alerts that are not important in a particular case may appear. Conversely, the absence of alerts does not guarantee there are no aspects of the results needing attention. It is up to the individual to critically assess their own results and, if necessary, seek expert advice.

Publication of your CIF in IUCr journals

A basic structural check has been run on your CIF. These basic checks will be run on all CIFs submitted for publication in IUCr journals (*Acta Crystallographica*, *Journal of Applied Crystallography*, *Journal of Synchrotron Radiation*); however, if you intend to submit to *Acta Crystallographica Section C* or *E* or *IUCrData*, you should make sure that full publication checks are run on the final version of your CIF prior to submission.

Publication of your CIF in other journals

Please refer to the *Notes for Authors* of the relevant journal for any special instructions relating to CIF submission.

PLATON version of 13/07/2021; check.def file version of 13/07/2021

Datablock pfc-73-cu - ellipsoid plot

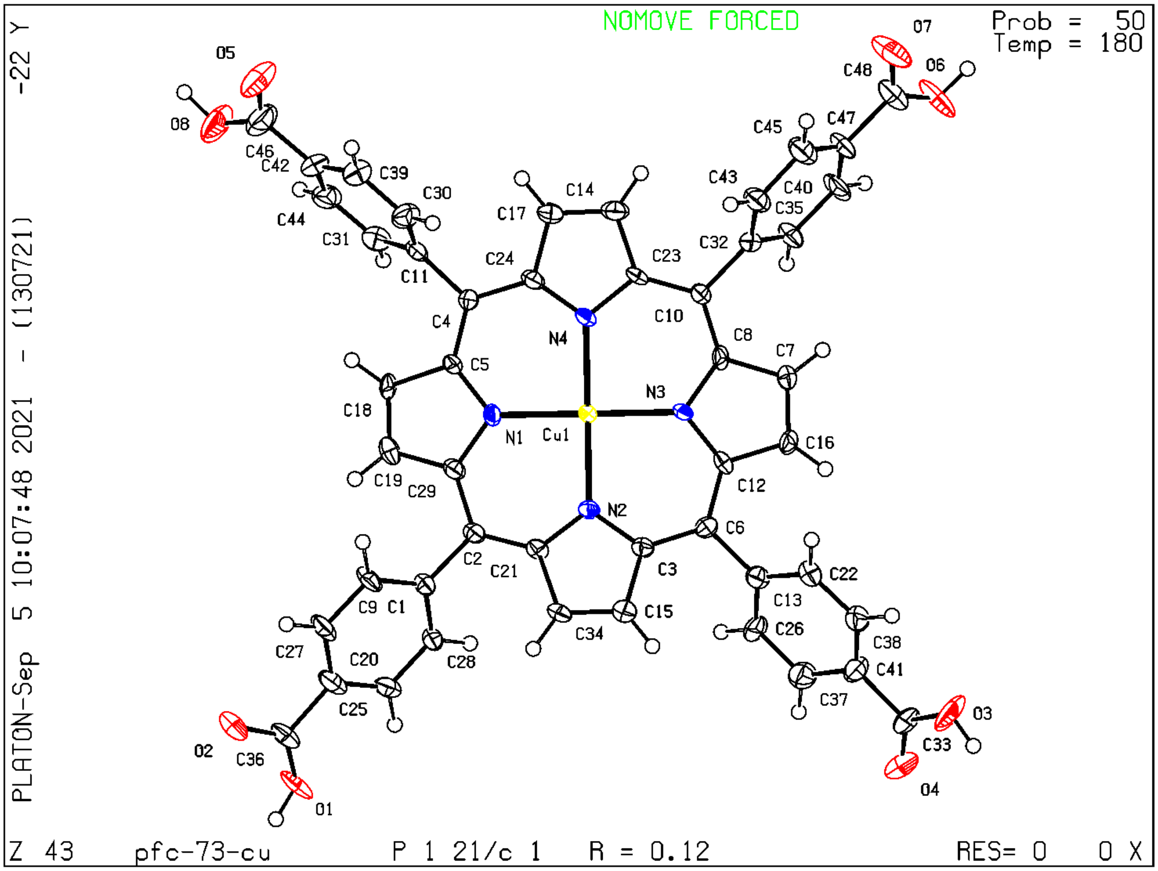

Supplement: Supplementary file 1 — Supplementary Information [file 41467_2022_30523_MOESM1_ESM.pdf]
